# Histopathology of the broad class of carbon nanotubes and nanofibers used or produced in U.S. facilities in a murine model

**DOI:** 10.1186/s12989-021-00440-z

**Published:** 2021-12-20

**Authors:** Kelly Fraser, Ann Hubbs, Naveena Yanamala, Robert R. Mercer, Todd A. Stueckle, Jake Jensen, Tracy Eye, Lori Battelli, Sidney Clingerman, Kara Fluharty, Tiana Dodd, Gary Casuccio, Kristin Bunker, Traci L. Lersch, Michael L. Kashon, Marlene Orandle, Matthew Dahm, Mary K. Schubauer-Berigan, Vamsi Kodali, Aaron Erdely

**Affiliations:** 1 Health Effect Laboratory Division, National Institute for Occupational Safety and Health, Pathology and Physiology Research Branch, 1095 Willowdale Rd, MS-2015, Morgantown, WV 26505-2888 USA; 2 West Virginia University, Morgantown, WV USA; 3 Division of Cardiovascular Disease and Hypertension, Rutgers Robert Wood Johnson Medical School, New Brunswick, NJ USA; 4 RJ Lee Group, Monroeville, PA USA; 5 Division of Field Studies Evaluation, National Institute for Occupational Safety and Health, Cincinnati, OH USA; 6 International Agency for Research On Cancer, Lyon, France

## Abstract

**Background:**

Multi-walled carbon nanotubes and nanofibers (CNT/F) have been previously investigated for their potential toxicities; however, comparative studies of the broad material class are lacking, especially those with a larger diameter. Additionally, computational modeling correlating physicochemical characteristics and toxicity outcomes have been infrequently employed, and it is unclear if all CNT/F confer similar toxicity, including histopathology changes such as pulmonary fibrosis. Male C57BL/6 mice were exposed to 40 µg of one of nine CNT/F (MW #1–7 and CNF #1–2) commonly found in exposure assessment studies of U.S. facilities with diameters ranging from 6 to 150 nm. Human fibroblasts (0–20 µg/ml) were used to assess the predictive value of in vitro to in vivo modeling systems.

**Results:**

All materials induced histopathology changes, although the types and magnitude of the changes varied. In general, the larger diameter MWs (MW #5–7, including Mitsui-7) and CNF #1 induced greater histopathology changes compared to MW #1 and #3 while MW #4 and CNF #2 were intermediate in effect. Differences in individual alveolar or bronchiolar outcomes and severity correlated with physical dimensions and how the materials agglomerated. Human fibroblast monocultures were found to be insufficient to fully replicate in vivo fibrosis outcomes suggesting in vitro predictive potential depends upon more advanced cell culture in vitro models. Pleural penetrations were observed more consistently in CNT/F with larger lengths and diameters.

**Conclusion:**

Physicochemical characteristics, notably nominal CNT/F dimension and agglomerate size, predicted histopathologic changes and enabled grouping of materials by their toxicity profiles. Particles of greater nominal tube length were generally associated with increased severity of histopathology outcomes. Larger particle lengths and agglomerates were associated with more severe bronchi/bronchiolar outcomes. Spherical agglomerated particles of smaller nominal tube dimension were linked to granulomatous inflammation while a mixture of smaller and larger dimensional CNT/F resulted in more severe alveolar injury.

**Supplementary Information:**

The online version contains supplementary material available at 10.1186/s12989-021-00440-z.

## Introduction

Carbon nanotubes and nanofibers (CNT/F) are known to cause pathologic changes in the lung, both in the alveolar and bronchiolar regions [1–6]. The fibrogenic and inflammatory capabilities of CNT/F, with reliance upon short-term and subacute animal studies that investigated pulmonary inflammation, granulomatous inflammation, and thickening of the alveolar septum, motivated the establishment of the National Institute for Occupational Safety and Health (NIOSH) recommended exposure limit (REL) for CNT/F [7]. NIOSH’s RELs are generated through quantitative risk assessment which characterize the exposure–response relationship of an identified hazard. The REL establishes an upper exposure limit based upon risk for an 8-h time-weighted average airborne concentration of a xenobiotic during a 40-h work week over a 45-year working lifetime and an analytical capability for measuring the agent at the concentration limit. Specifically, for CNT/F, the NIOSH REL was established as 1 µg/m^3^ for respirable mass of elemental carbon with the additional recommendation that CNT/F exposures should be controlled as much as possible below 1 µg/m^3^ [7].

The broad class of CNT/F exhibit a vast array of physicochemical characteristics including an extensive range of diameters and lengths in conjunction with functionalization, surface coatings, etc., which are critical for the applications of these particles for their engineered purposes, yet are also critical for driving the toxicity of CNT/F. In general, comparative studies of CNT with different characteristics identified variable toxicity consistent with the fiber paradigm. CNT/F with longer lengths and diameters were mostly found to induce greater inflammation and injury than thinner and shorter CNT/F [2, 4, 8–20]. The longer and thicker CNT/F, or rod-like materials, were more likely to impact non-cell mediated translocation and airway fibrosis compared to spherical agglomerating CNT/F [2, 3, 20–22]. However, this pattern was not entirely consistent as noted in several studies. CNT/F with various physical dimension characteristics were previously found to induce similar genotoxicity, as assessed by micronuclei formation, and small agglomerated and rod-like CNT both induced carcinogenesis [20, 23, 24]. Also, single-walled CNT, by evading macrophage recognition, was found to induce greater alveolar fibrillary collagen production than a rod-like CNT [3]. Lastly, in addition to particle nominal tube dimensions, surface functionalization and coating have the potential to either increase or decrease toxicity by affecting cellular interactions [4, 25–27].

Detailed exposure assessments over the years have confirmed a wide spectrum of particle characteristics in the workplace and integrated exposure and toxicity assessments have indicated that not all CNT/F confer similar toxicity [4, 20, 28–30]. An area of increased focus is the inhalable fraction, the particle fraction that contains CNT/F which enters the respiratory tract and can deposit in the conducting airways [31]. In vivo studies indicated bronchiolar fibrosis and bronchiolitis obliterans-like changes as a potential outcome in experimental exposures [2, 4]. Human health effect studies also indicated that the inhalable fraction more closely associated with measured responses compared to assessment of the respirable fraction only, the subfraction of the inhalable particles that enter the alveolar region of the lung [32–35]. These advancements in knowledge since the publication of the NIOSH CNT/F Current Intelligence Bulletin in 2013 necessitate studies comparing the broad spectrum of CNT/F with more emphasis on the potential effects on the bronchiolar region, or the inhalable fraction and whether all CNT/F should be considered equally.

To date, the simultaneous characterization of the pathological changes of the broad class of CNT/F used or produced in U.S. facilities is lacking. These changes, such as fibrosis, are critical as they represent fundamental and potentially irreversible changes in the lung [2, 3, 6, 9, 22]. The aim of the present study was to generate a toxicity profile of pathology for a series of CNT/F of various physicochemical characteristics. Emphasis was placed on discriminating between alveolar and bronchiolar effects given the association of the inhalable fraction with human health responses and the REL currently only considering the respirable fraction. The in vivo studies were complemented with in vitro comparability of human fibroblast cells. Lastly, computational modeling was applied to determine which physicochemical characteristics corresponded to which pathological outcome and how the various CNT/F grouped.

## Results and discussion

**Fig. 1:**
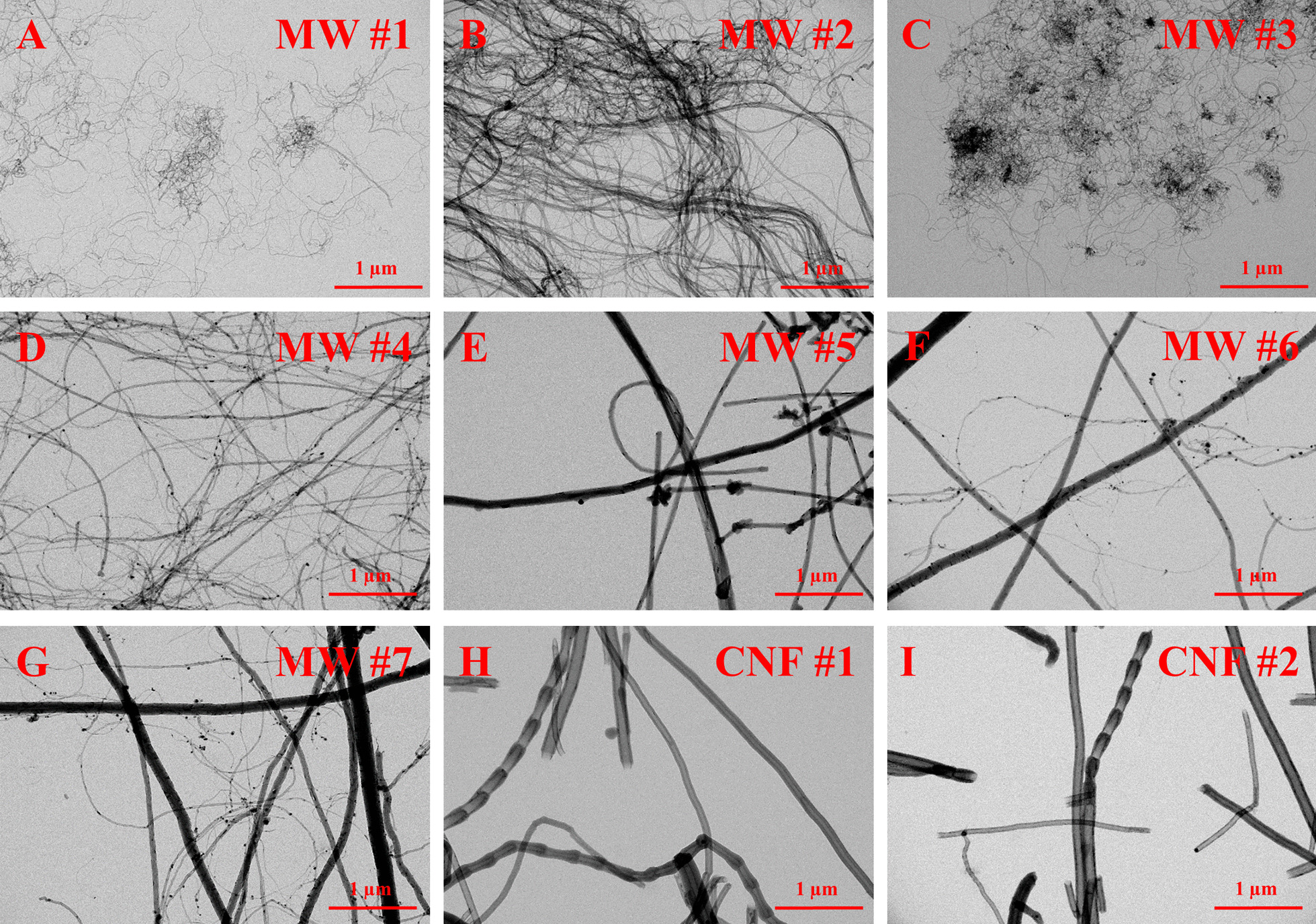
Representative TEM images of particle dispersed in isopropanol. Scale bar represents 1 µm. Images A-I are of MW #1–#7, and CNF #1–#2 respectively. Materials of small diameter and length (MW #1, #3) were spherical agglomerates. MW #2 is a unique material that forms elongated aggregates. As materials increase in diameter and length, the agglomeration becomes less, and singlets are more common. Some materials, including MW #6 and #7, have mixed populations

The nine materials used in this study were selected to represent the broad class of CNT/F [30]. In brief, seven multi-walled carbon nanotubes (MWCNT) and two carbon nanofibers (CNF) were numbered in order of their diameter as reported by the production facility and are referred to as MW #1–7 and CNF #1–2 (Fig. 1). MWCNT-7/Mitsui-7, labeled as MW #5 in this study, was used as a benchmark material for comparison. Analyses and interpretation of detailed physicochemical characteristics and genotoxicity were presented separately [20]. In this section of the evaluation of CNT/F toxicities, in vivo assessments for pathological changes were compared and correlated to physicochemical characteristics. Male C57BL/6 J mice were exposed to 40 µg of CNT/F or dispersion medium (DM) via oropharyngeal aspiration and euthanized at 84 d post-exposure. Historically, a 40 µg dose of CNT/F was necessary to induce pathological changes within an 84 d post-exposure time point and induce significant alveolar fibrosis [3, 4, 6, 22]. Rodent lungs were gravity filled with formalin to fix the tissue and preserve inflation to evaluate particle deposition, alveolar fibrosis, and other histopathology changes.

Additional in vitro assessments were used for comparison and to further investigate the mechanisms by which the pathological changes occurred. For comparison, human fibroblast cells were cultured and exposed to 0–20 µg/ml of each of the nine CNT/F for 24 and 48 h and subsequently assessed for cell viability and proliferation as well as collagen-1, α-smooth muscle actin, and TGF-β1 production.

### Particle characterization

The CNT/F used in this study were extensively characterized in a previous publication and can be found in the Additional file 1 (Additional file 1: Tables S1–S3, Additional file 1: Figs. S1 and S2) [20]. Representative micrographs from transmission electron microscopy (TEM) images of each particle are shown in Fig. 1. It was quite evident that as CNT/F increase in nominal tube diameter and length, bundled agglomeration generally decreased with a transition point at the characteristics of MW #4. MW #1 and #3 were predominately spherical agglomerates with a geometric mean under 1 µm (Additional file 1: Table S2). MW #2 was a unique highly entangled cross-linked MWCNT as previously described [4, 20] that forms two separate populations of loosely bundled agglomerates and extensive ‘rivers’ of aggregates, and agglomerates of aggregates, in the micron size range (Fig. 1; Additional file 1: Table S2). MW #4–7 and CNF #1–2 were almost exclusively bundled agglomerates exceeding a 3:1 aspect ratio (≥ 96%; Additional file 1: Table S2). The analysis of physical characteristics may also encompass assumptions of CNT/F rigidity. While some studies have attempted to correlate rigidity to toxicity outcomes, the measurement is limited due to the lack of current universal standards of measurement for CNT/F and was not performed [36, 37].

### Histopathology

For comparative potency between materials, 40 µg CNT/F is a known administered dose to induce pathology including fibrillary collagen thickening of alveolar septa. Previously, we indicated this dose to likely exceed a lifetime exposure at average exposure levels in U.S. facilities [29, 38] and our human health effect studies supported our extrapolations [32–34]. The goal of this study was less concerned with deposited dose with relation to human equivalency as compared to relative potency. Combined with the known fact that MW #5 (Mitsui-7/MWCNT-7) does not induce a significant alveolar fibrosis up to 20 µg, the dose of 40 µg was selected to be able to evaluate differences of effect between the nine CNT/F [3, 22].

**Fig. 2:**
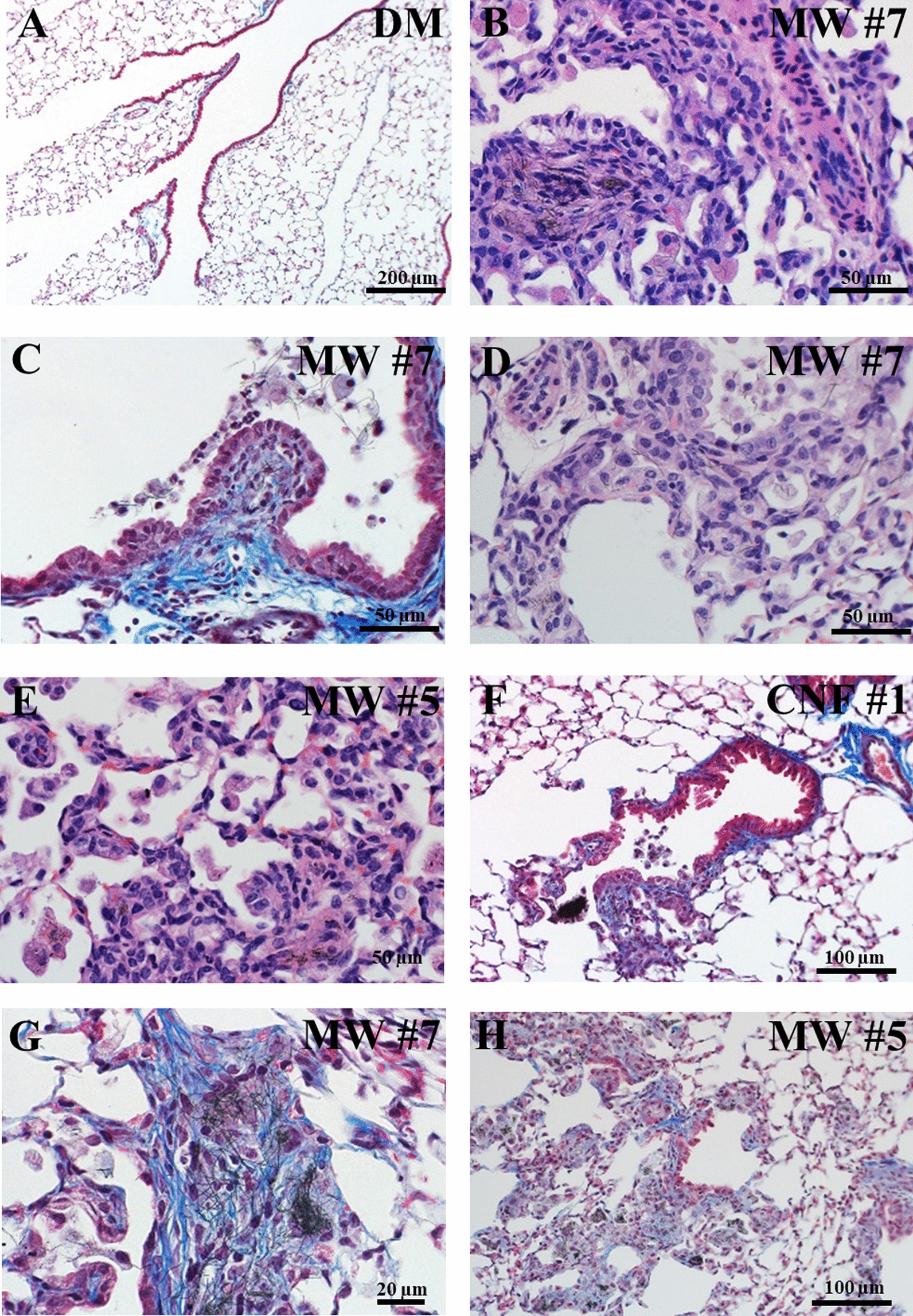
Representative micrographs at 84 d post-exposure of pathologies seen following CNT/F exposure: **A** DM exposed mouse, **B** granulomatous bronchopneumonia, **C** bronchiolitis obliterans-like changes, **D** alveolar histiocytosis, hypertrophy, and hyperplasia, **E** alveolar epithelial cell hypertrophy and hyperplasia, alveolar histiocytosis, and interstitial fibrosis, **F** bronchiolar epithelium hypertrophy, and bronchiolar fibrosis **G**, bronchiolar fibrosis, and **H** alveolar interstitial fibrosis

At 84 d post-exposure, CNT/F often caused granulomatous inflammation, defined as an organized infiltrate of epithelioid macrophages which may form giant cells and are often admixed with lymphocytes, plasma cells, and fibrosis. Diagnoses within this category included granulomatous bronchopneumonia, but also included histiocytic bronchopneumonia, granulomatous bronchointerstitial pneumonia, lymphogranulomatous interstitial pneumonia, and granulomatous alveolitis. An example image can be found in Fig. 2B. The distribution and severity of the granulomatous response was significant among all treatment groups (Table 1).

**Table 1:**
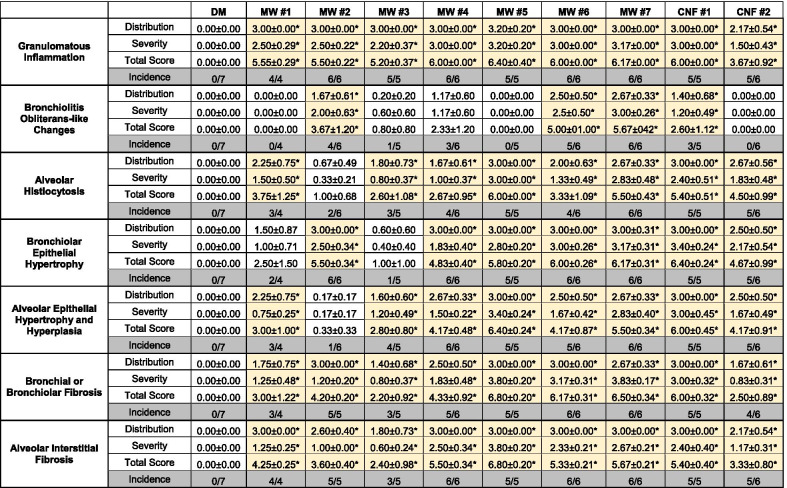
Histopathology scoring for severity, distribution, total score, and incidence

*Indicates *p* < 0.05 as significantly different from control

Morphologic responses to some CNT/F included proliferative changes within airways that sometimes obstructed the lumen bronchioles and alveolar ducts. These were classified as proliferative bronchiolitis obliterans (Fig. 2C) when bronchioles were affected. If the alveolar ducts were involved, these were classified as bronchiolitis obliterans-like lesions of the alveolar duct. These potentially obstructive lesions often formed in association with granulomatous inflammation and projected into the lumen of bronchioles and alveolar ducts. MW #1, 5 and CNF #2 did not induce proliferative bronchiolitis obliterans or bronchiolitis obliterans-like morphologic alterations and the incidence was only 1/5 for MW #3 (Table 1). MW #4 had incidence in half the analyzed lungs, but significance was not reached in terms of severity and distribution. MW #2, 6, 7, and CNF #1 induced significant bronchiolitis obliterans-like changes (Table 1). We have previously shown proliferative bronchiolitis obliterans lesions to occur following MW #2 exposure consistent with the large aggregates [4].

In the alveolar region of the lungs, mice exposed to CNT/F often had accumulations of alveolar macrophages, a finding also known as alveolar histiocytosis (Fig. 2D). In some cases, alveoli with alveolar macrophage accumulation also showed evidence of a tissue response (histiocytic alveolitis) and/or neutrophils accompanied the macrophages (histiocytic and neutrophilic alveolitis). Because the responses represented a spectrum of predominantly histiocytic responses, these diagnoses were grouped together in the summary table. All CNT/F induced alveolar histiocytosis except for MW #2 (Table 1), the CNT with larger aggregates and agglomerates that has notably less alveolar deposition compared to other CNT/F [4].

Exposed mice also developed hypertrophy of the bronchiolar epithelium as well as alveolar epithelial hypertrophy and hyperplasia (Table 1; Fig. 2E, F). Particle deposition and persistence were necessary for bronchiolar hypertrophy and alveolar hypertrophy and hyperplasia. MW #1 and #3 did not induce bronchiolar epithelial hypertrophy while MW #2 did not induce alveolar epithelial hypertrophy and hyperplasia. MW #4–7 and CNF #1–2 had significant effects for both. Bronchial/bronchiolar and alveolar interstitial fibrosis varied in frequency and severity in a CNT/F related manner (Table 1, Fig. 2F–H) as detailed in the next sections.

**Fig. 3:**
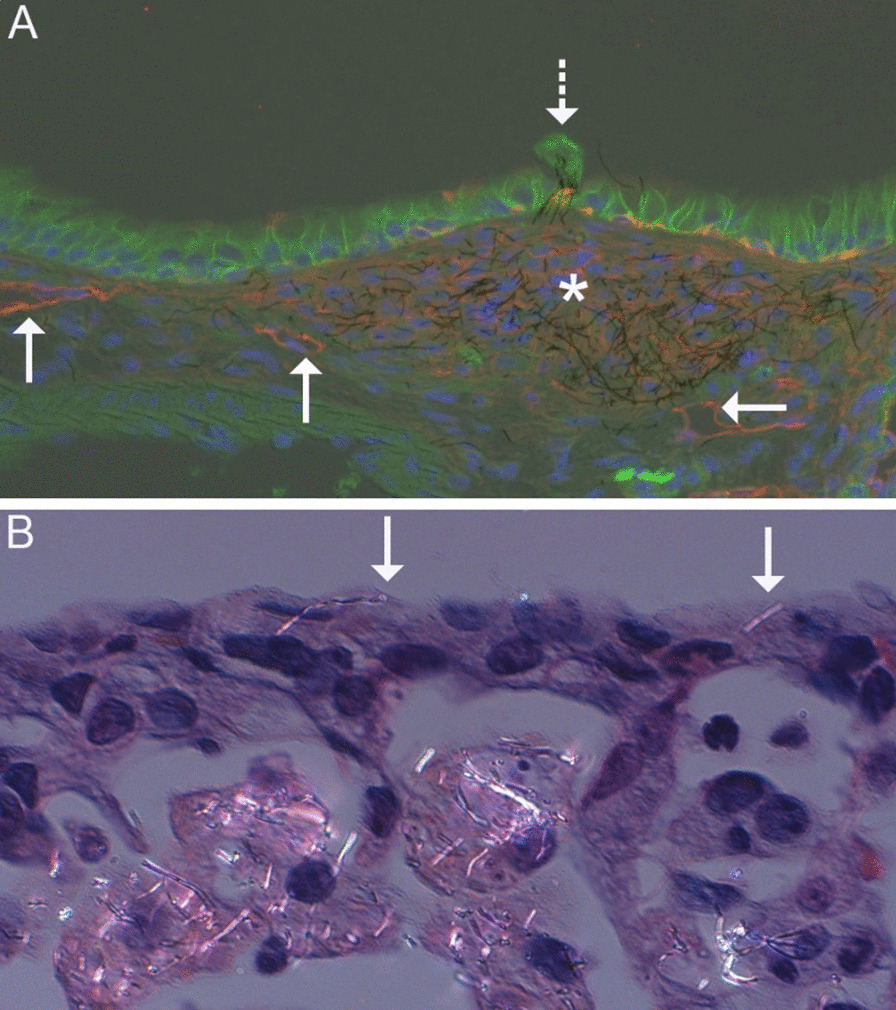
Localization of CNT/F in relation to the lymphatics and pleura. **A** Immunofluorescent image of a bronchiole from a mouse 84 d after MW #6 exposure showing staining for e-cadherin (green) and podoplanin (red). Podoplanin was present in lymphatic endothelium, alveolar type 1 cells, and basal cells and allows the demonstration of bronchiolar lymphatics (solid arrows) and a meshwork of podoplanin staining consistent with lymphangiogenesis in the bronchiolar wall (*). MW #6 was present within that meshwork and a few MW extend from the meshwork through the epithelium (dashed arrow). **B** Photomicrograph of a partially polarized lung section from a mouse 84 d after CNF #1 exposure. CNF were present in alveolar macrophages and two CNF were in the pleura (arrows)

Additional morphologic changes regarding lymphatics are of note. The lymphatics of the lung control interstitial fluid balance, transport cells of the immune system, and participate in particle clearance in the lung [39–41]. In this study, particle accumulation, particularly in materials of longer length, could be found at the bronchoalveolar junction, the location of some of the smallest lymphatic vessels (Fig. 2G). This accumulation has the potential to obstruct lymphatic flow and may be a mechanism for decreased clearance in fibrotic and inflamed airways. Similarly, needle-like particles can damage macrophages attempting to reach lymphatic capillaries surrounding the terminal bronchioles resulting in further particle accumulation. Even when unobstructed by previous particle deposition, clearance of long, somewhat rigid particles may be difficult in the narrow and curving lymphatic pathways. Ectasia, or dilation, of lymphatic vessels was observed as was also previously reported following MWCNT exposure [6]. It remains to be determined whether pleural accumulation of CNT/F and the airway fibrosis were attributable to altered lymphatic clearance. However, in some cases clusters of CNT/F could be seen extending from dilated lymphatics or foci of presumptive lymphangiogenesis (new lymphatic formation) through the airway wall to the epithelial surface of airways suggest lymphatic obstruction and potential release of CNT/F back into airways (Fig. 3A). Furthermore, lymphatics are not restricted to the pulmonary lobule, but are also present in interlobular septa and the pleura [41]. Macrophages containing particles often traffic to the lymphatics to clear particles from the lung [40]. Additionally, macrophage-mediated transport of CNT/F to pleural lymphatics can potentially release CNT/F near the pleural lining if the particles are cytotoxic. Multiple incidences of pleural penetration or accumulation at the pleural surface were seen following exposure to MW #5–6 and CNF #1–2 (Fig. 3B). However, more sensitive techniques for nanoparticle detection in tissues, such as enhanced darkfield microscopy, might enhance demonstration of pleural penetrations. MW #5 has been previously reported to penetrate the pleura, though it is unclear if all particles, particularly spherical agglomerates (e.g., MW #1 and #3) may penetrate the visceral pleura [6].

### Histopathologic assessment of pulmonary fibrosis

Two sites of fibrosis in the lung, bronchial/bronchiolar and alveolar (Table 1, Fig. 2F–H), were semi-quantitatively evaluated for distribution (none = 0, focal = 1, locally extensive = 2, multifocal = 3, multifocal and coalescent = 4, or diffuse = 5) and severity (none = 0, minimal = 1, mild = 2, moderate = 3, marked = 4, and severe = 5). Bronchial and bronchiolar fibrosis occurred frequently in conjunction with bronchiolitis obliterans-like changes and/or granulomatous inflammation. Incidence of 100% was observed for MW #5–7 and CNF #1. MW #2 also had 100% incidence given the nature of the highly entangled particles that deposit significantly in the conducting airways [4]. MW #1, #3, and CNF #2, although not without effect, had lower severity and distribution scores. Histopathologic assessment of alveolar interstitial fibrosis indicated greater severity and distribution with MW #4–7 and CNF #1 as compared to other materials, especially MW #3 and CNF #2 (Table 1).

### Morphometric analysis of pulmonary fibrosis

**Fig. 4:**
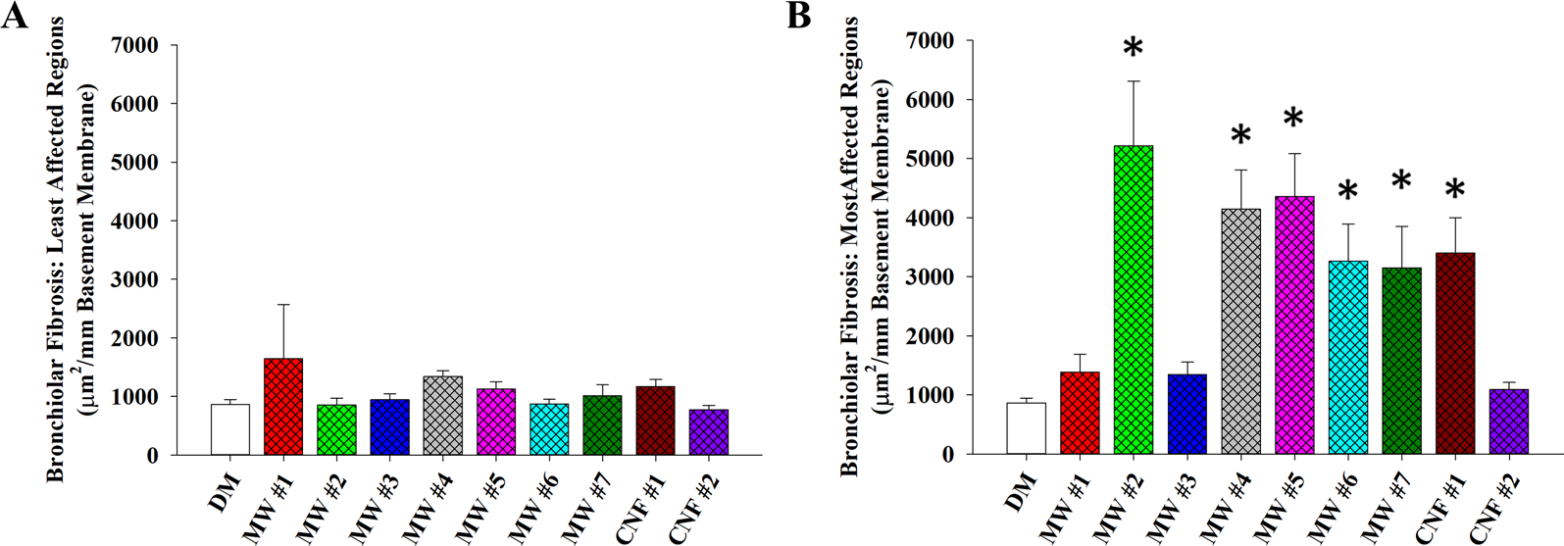
Bronchiolar fibrosis at 84 d post-exposure to 40 µg dose of CNT/F. The total area of bronchial/bronchiolar fibrosis per mm of basement membrane was quantified in two regions, both the least affected (**A**) and most affected (**B**) regions for comparison. *p < 0.05 versus DM. Scale bars represent mean ± standard error

To further investigate pulmonary fibrosis, bronchiolar and alveolar fibrosis was quantitatively evaluated by morphometry. To measure fibrosis in the terminal bronchioles, photomicrographs were taken of trichrome-stained lung sections. Using lung sections stain with Masson’s trichrome stain and ImageJ software (ImageJ (nih.gov)), measurements were (1) the area of collagen staining in the wall of the terminal bronchiole, (2) the total area of the region evaluated, and (3) the length of the basement membrane for the area measured. This enabled calculation of the area of fibrosis per micron of basement membrane (Fig. 4) and as percent area of fibrosis per area of the total field of view (Additional file 1: Fig. S3). Two distinct regions were systematically sampled in each lung section: (1) areas of minimal to no severity, or least affected terminal bronchioles, and (2) areas of most severe fibrosis, or most affected terminal bronchioles. In the least affected areas, no significant differences were seen from control (Fig. 4A). For the most affected regions, a significant increase in bronchiolar fibrosis was measured in MW #2, 4–7 and CNF #1 exposed lung tissue, but not for MW #1, 3, or CNF #2 (Fig. 4B). Fibrosis was consistently observed in areas of prominent particle deposition, and notable in areas with particle agglomerates. Similar findings were reported by Duke et al., in which they quantified the area to perimeter ratio of airway fibrosis and found that “rod-like” MWCNT that are similar in morphology to MW #5–7 and CNF #1–2 of the present study were more likely to induce greater airway fibrosis than “tangled” MWCNT similar to MW # 1 and #3 in the present study [42]. These differences in airway fibrosis were attributed to variability in clearance mechanisms and the translocation of “rod-like” particles across the epithelium where pathways driving fibroblast activity can be initiated. Interestingly, CNF #2, while having a diameter more like MW #5–7 and CNF #1, did not induce significant bronchiolar fibrosis as measured by morphometry. This may be due to the smaller nominal tube lengths (Additional file 1: Table S1; Additional file 1: Fig. S2). These findings support the conclusion that the physical presence of particle deposition in airways, with physical dimensions of greater diameter and length, act as a potent driver of airway fibrosis.

**Fig. 5:**
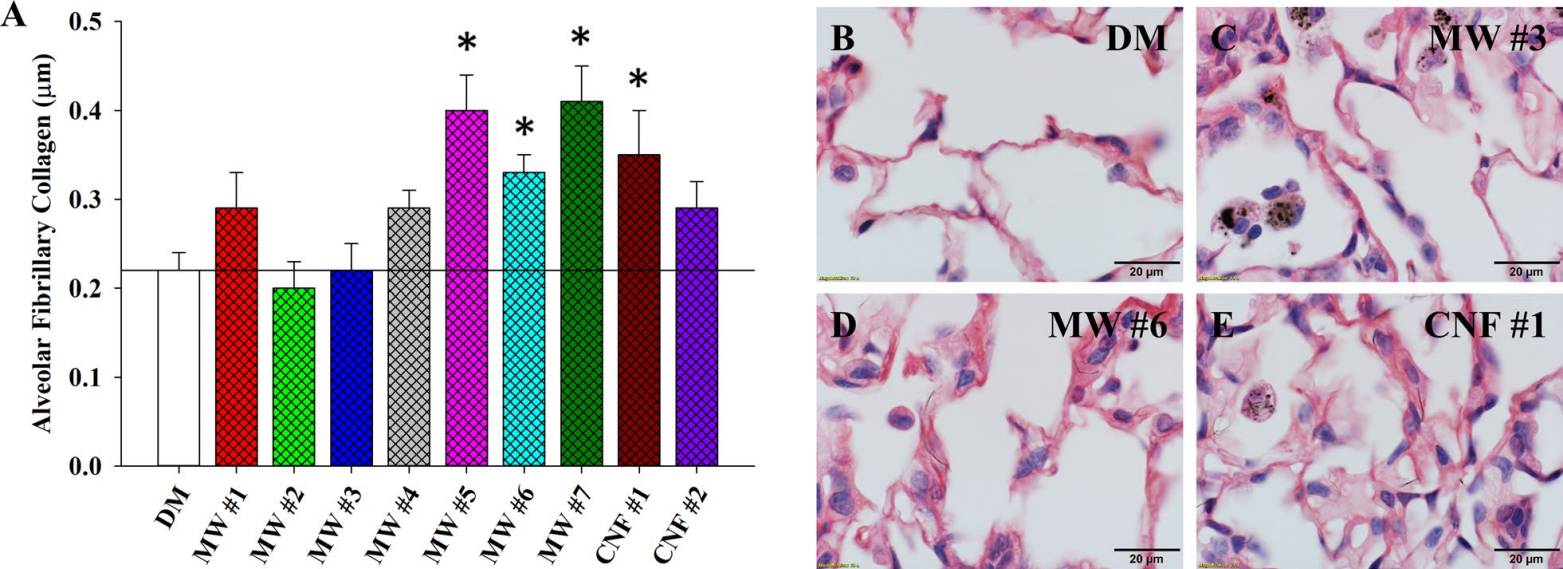
Alveolar fibrillary collagen thickness quantification. **A** Alveolar fibrillary collagen thickness 84 d post-exposure to CNT/F. Representative micrographs of Picrosirius red stained tissue were as follows: **B** DM, **C** MW #3, **D** MW #6, and **E** CNF #1. Particle is visibly present in the interstitial walls in **D** and **E**. *p < 0.05 versus DM. Scale bars represent mean ± standard error

Alveolar fibrosis is a well-documented outcome following CNT exposure [3, 6, 22]. Morphometric point and intercept counting was used to measure the thickness of alveolar fibrillary collagen in Sirius Red stained lung sections at 84 d post-exposure. Fibrillary collagen was significantly increased in mice exposed to MW #5–7 and CNF #1 (Fig. 5A). Representative micrographs of vehicle-exposed as well as MW #3, #6, and CNF #1 can be found in Fig. 5B–E respectively. Areas of fibrosis were related to the presence of particle in the interstitium. The increased alveolar fibrillary collagen thickness of MW #5 exposed lungs was consistent with previous investigations [3, 22]. Also, it should be noted that increased alveolar fibrillary collagen thickness for MW #5 was similar whether the route of exposure was by inhalation or oropharyngeal aspiration, the method of exposure used in this study. Increases were found for MW #1, MW #4, and CNF #2 but significance was not achieved, suggesting: (1) these particles would likely increase alveolar fibrillary collagen thickness at deposited levels greater than 40 µg and (2) the nominal tubes per unit mass were at an insufficient number displaying the key physicochemical characteristic(s) needed to induce significance. Given we extrapolate 40 µg in a mouse to exceed a normal lifetime deposited dose in a human exposed under current recommended workplace exposure limits, these materials may be less likely to result in interstitial fibrosis in workers. Mercer et al., compared single-walled (SW) CNT and MWCNT rodent lung aspiration exposures and reported that macrophages were less likely to recognize and phagocytose SWCNT compared to MWCNT, leading to greater interstitial accumulation and fibrosis from SWCNT than MWCNT [3]. As the size of MW #1 in the current study approaches the size of SWCNT, it is suspected that the infrequent singlet or very small agglomerates may escape macrophage recognition permitting translocation to the alveolar interstitium and induction of interstitial fibrosis, therefore behaving in a similar fashion to SWCNT resulting in the increase in interstitial fibrosis, although not reaching statistical significance in this study. There was no effect for MW #2 or #3. This was expected for MW #2 given the sample is less likely to accumulate in the alveolar region [4]. Of note, several instances were observed, particularly in MW #3 exposed lung tissue, where small agglomerates of particles were present in the interstitium, but fibrosis was not present, suggesting that particle singlets were more likely to induce fibrosis than tangled agglomerated particles (Fig. 5C).

### Hierarchical clustering analysis (HCA) and principal component analysis (PCA) of the histopathology outcomes

**Fig. 6:**
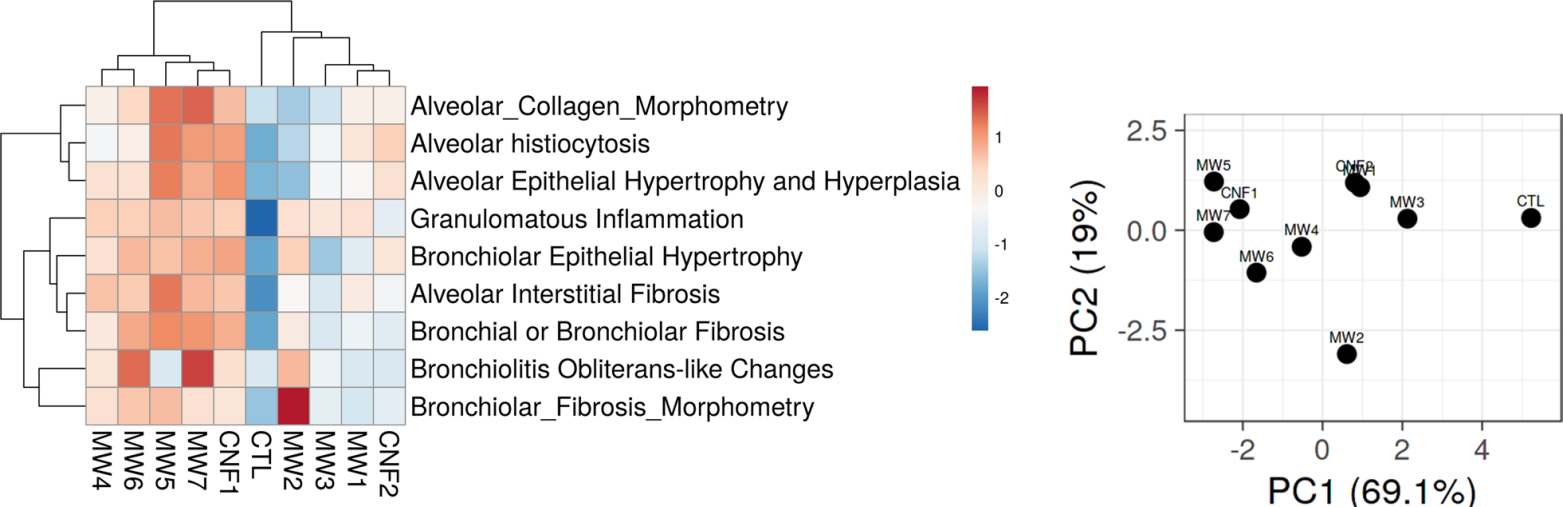
Hierarchical clustering analysis (HCA) and principal component analysis (PCA) of different CNT/F materials comparing all histopathology outcomes. For the PC, distance between two materials reflects the proximity in outcomes between them

**Fig. 7:**
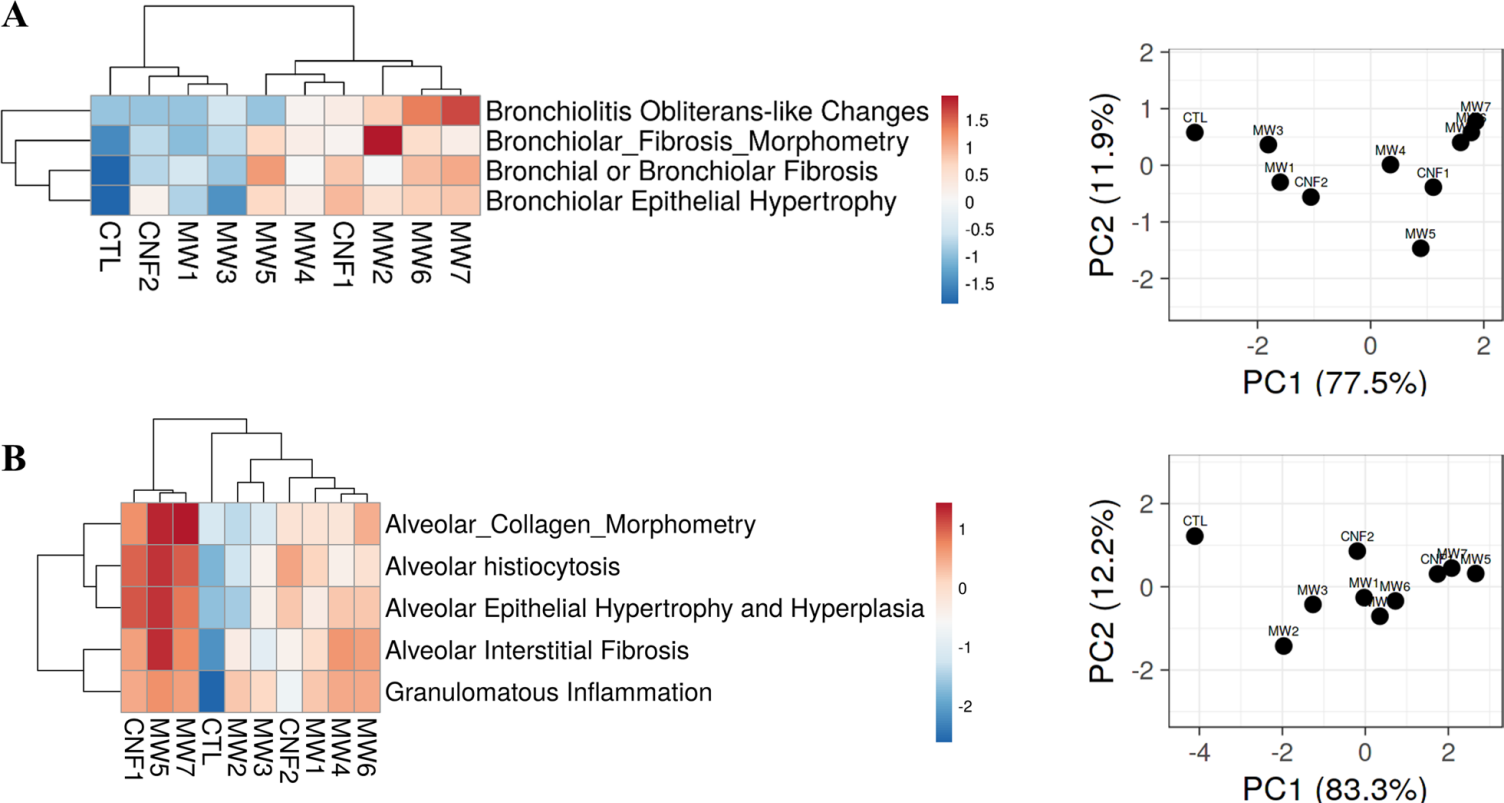
Hierarchical clustering analysis (HCA) and principal component analysis (PCA) of different CNT/F materials and bronchi/bronchiolar histopathology outcomes (**A**) and alveolar histopathology outcomes (**B**). For the PC, distance between two materials reflects the proximity in outcomes between them

HCA and PCA were performed to group CNT/F by adverse histopathology. Initially all outcomes assessed were analyzed (Fig. 6). Overall, severity of response was greater for MW #4–7 and CNF #1 as compared to MW #1–3 and CNF #2 which grouped more closely with the sham group (DM, CTL). These results were intriguing as the lesser responding grouping included a large nominal diameter material in CNF #2 as well as MW #2 which primarily has bronchiolar effects. To segregate regional effects, the alveolar and bronchiolar responses were evaluated separately. The bronchiolar effects were like the overall response except for MW #2 which corresponded in response more closely with MW #6 and #7 (Fig. 7A). MW #1, #3, and CNF #2 localized with the sham (DM, CTL) exposed mice indicating minimal to no bronchiolar effects. The analysis to address specifically the alveolar outcomes indicated that MW#5, #7, and CNF #1 had significant effects compared with the remaining materials (Fig. 7B). These three materials segregated out as they induced marked responses in all five alveolar assessments (Fig. 6, Table 1). The other materials had variable significance in alveolar responses. MW #2 and #3 induced milder responses in general with the exception of granulomatous inflammation and MW #4 and #6 had generally stronger effects than MW #2 and #3 but less than MW #5, #7, and CNF #1 (Fig. 7B, Table 1). In summary MW #5, #7 and CNF #1 overall induced the strongest with effects in both the bronchiolar and alveolar regions hence their close clustering in Fig. 6. MW #4 and MW #6 were positive in effect for bronchiolar and alveolar effects but generally milder compared to MW #5, #7 and CNF #1. MW #1–3 and CNF #2 generally induced milder or specific regional effects as was the case with MW #2 in the bronchiolar region.

### HCA and PCA of the histopathology outcomes with physicochemical characteristics

**Fig. 8:**
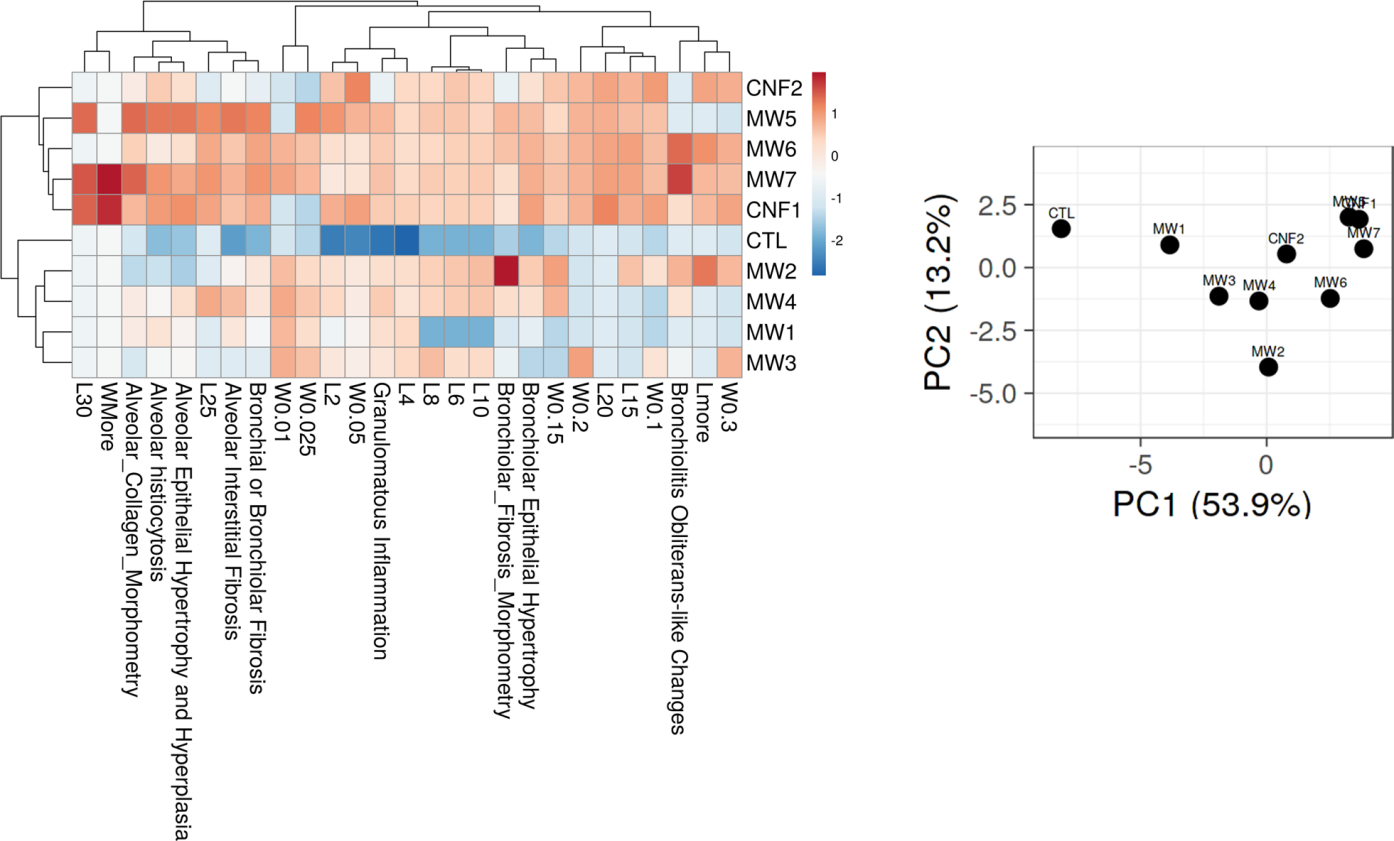
Clustering of physical dimension length (L) and width (W) binning of CNT/F materials with all histopathology outcomes as hierarchical clustering analysis and principal component analysis

**Fig. 9:**
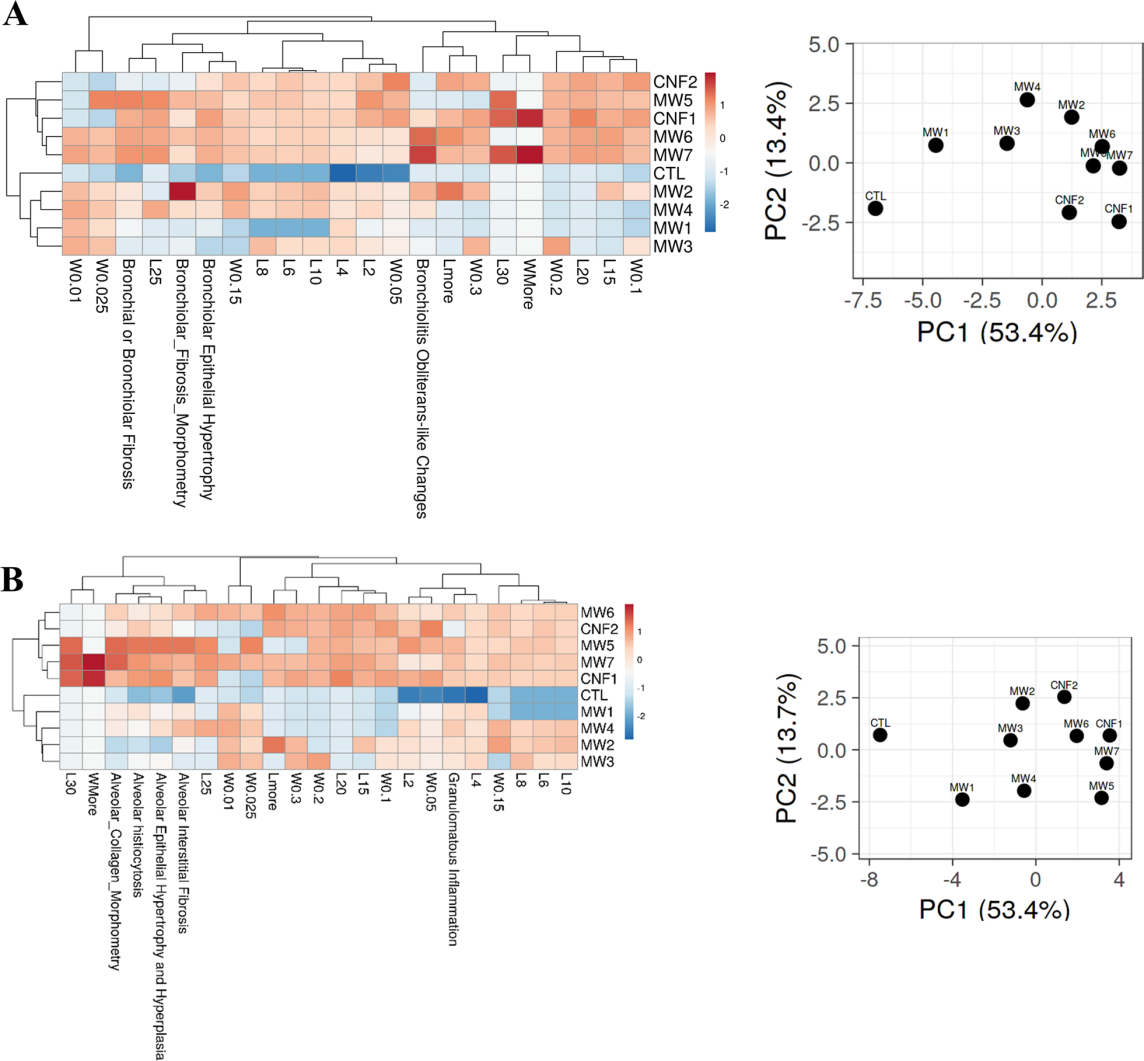
Clustering of physical dimension length (L) and width (W) binning of CNT/F materials with bronchial/bronchiolar histopathology outcomes (**A**) and alveolar pathology outcomes (**B**)

The next step was to include the physicochemical characteristics with the histopathology outcomes in additional analyses to provide inference into specific characteristics that contributes to developing pathology. The analysis was done in three ways: (1) detailed characterization of binned length (L) and diameter/width (labeled as W for figure clarity for easier distinction from L); (2) standard physicochemical data using means only from Additional file 1: Tables S1–S3; and (3) the combination of binned length and diameter/width and means only data. The data corresponding to all pathology outcomes as well as bronchiolar and alveolar separately were considered for analysis. Previously, using computational modeling, we have shown that the binned length and diameter provided the resolution to accurately group CNT/F in comparison to using means only [20]. In fact, the grouping using binned length and diameter alone was comparable to extensive characterization of all physicochemical parameters. For the histopathological outcomes in this study, adding the physicochemical characteristics did not exactly group outcomes alone as a separate cluster, but were clustered together with various physicochemical characteristics representative of the difference CNT/F materials investigated. Variations of MW #1–4 were typically in the lower responding group with remaining materials in the more toxic group (Figs. 8, 9; Additional file 1: Figs. S4–S9). The analysis of outcomes with physicochemical characteristics indicate that MW #1 and #3 consistently are of lower toxicity compared to MW #5–7 and CNF #1. MW #4 and CNF #2 represent a transition in toxicity from less to greater severity.

For alveolar pathology responses, in general all analyses highlighted associations with length, the larger length bins, and the agglomeration state (per_bundle_agglomerate_singlet, bundle_agglomerate_mean_length, bundled_agglomerate_mean_diameter). These results indicate that materials which contain nominal tubes with larger lengths, which were more prevalent for MW #5, #7, and CNF #1 (Additional file 1: Fig. S2) could induce more significant injury to the alveolar region. This was consistent with the material grouping for alveolar effects alone (Fig. 7B) and with physical dimension binning (Fig. 9B). The two-dimensional sizing was also a key factor in predicting alveolar pathology. MW #1 and #3 clearly form more spherical agglomerates (Additional file 1: Table S2). MW #4 was the transition point switching from majority spherical agglomerates to bundled/elongated agglomerates. The switch from spherical agglomerates to more elongated agglomerates and singlets is driven by increasing physical size. Therefore, as increasing length corresponds more with general severity of alveolar pathology, association with the transition away from spherical agglomerates (e.g., MW #1 and MW #3) was consistent. The inclusion of binning of the physical dimensions was able to better separate granulomatous inflammation from the rest of the alveolar-associated pathology with an association of the L2, L4, and W0.05 bins (Figs. 8, 9B). As all materials induced granulomatous inflammation (Table 1), correspondence with the smaller length bins, something that was consistent for all samples (Additional file 1: Fig. S2), were the associated drivers. Using means only (Additional file 1: Figs. S7 and S9), the association of granulomatous inflammation and alveolar interstitial fibrosis did not provide associations with any physical dimensions of length or width.

For bronchiolar pathology responses, the primary physicochemical characteristics driving the four measured outcomes were bundle_agglomerate_mean_length as well as longer lengths and wider diameters (e.g., L25, L30, and W0.15). This indicates a combination of increasing length and width, with a larger bundle_agglomerate_mean_length (e.g., MW #6 and #7) may have conferred greater bronchiolar toxicity. The analysis of bronchiolar effects alone grouped MW #1, MW #3, and CNF #2 with the sham (DM, CTL) group suggested a diminished response. This was intriguing given CNF #2 is a larger diameter material. Looking in more detail at the physicochemical characteristics, CNF #2, while having an increased diameter, did not have the corresponding increased length, especially in comparison to CNF #1 (Additional file 1: Figs. S1 and S2; Additional file 1: Table S1). This was further illustrated for the bundle_agglomerate_mean_length for CNF #2 that was closer to MW #1 than CNF #1 (Additional file 1: Table S2). Overall, increasing length and diameter, corresponding to bundled agglomerate that are larger than a 3:1 ratio conferred greater bronchiolar toxicity which can be stratified as MW #6 and MW #7 > MW #4, MW #5, and CNF #1 > MW #1, MW #3, and CNF #2. We confirmed the conducting airway effects of MW #2 as the larger of the two subpopulations of aggregates/agglomerates was too large to reach the alveolar space and had the largest bundle_agglomerate_mean_length. Also, the L2, L4, and W0.05 bins did not correspond to any bronchiolar effects (Figs. 8, 9A), which was consistent with those factors primarily driving alveolar granulomatous inflammation.

### In vitro fibrosis assessments

**Fig. 10:**
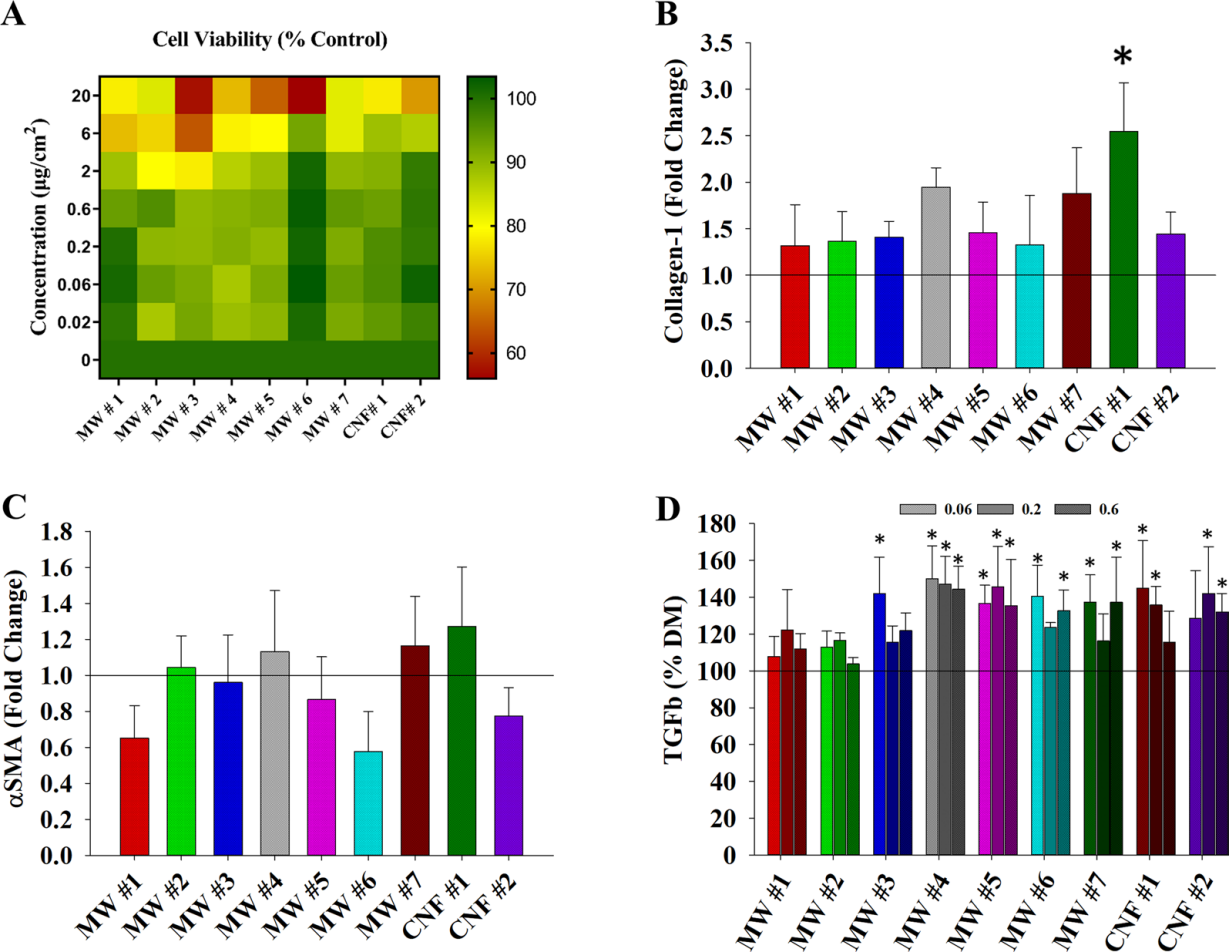
Human fibroblast in vitro cell viability (**A**), collagen-1 production (**B**), α smooth muscle actin production (**C**), and TGFβ1 secretion (**D**) following treatment with CNT/F at 0–20 µg/cm^3^ for 24 h. *p < 0.05 versus DM. Scale bars represent mean ± standard error

Human fibroblasts were used in this study to investigate the mechanisms driving fibrosis outcomes observed in vivo and to explore the efficacy of using in vitro single cell assays to model in vivo histopathological outcomes [43, 44]. The same nine CNT/F were used at concentrations of 0–20 µg/ml ranging from occupationally-relevant to overtly high doses in order to observe the full range of potential effects. Unlike epithelial cells, fibroblasts do not constitute the alveolar surface so estimates to in vivo deposited doses are difficult and were not extrapolated. Using a WST-1 assay, the percentage of cell viability and proliferation was quantified (Fig. 10A). The doses (up to 0.2 µg/ml) used for evaluation did not exert overt toxicity.

Fibroblasts are the key generators of collagen-1, the main collagen isoform comprising pulmonary fibrosis. Previous investigations showed that CNT penetrate to the interstitium, thus increasing the potential direct stimulation of fibroblasts to produce collagen [3, 22, 43–45]. Western blot was used to quantify collagen-1 production following 0.06 µg/ml exposure. Collagen-1 outputs were variable, although CNF #1 did induce a significant increase (Fig. 10B). Additionally, α-smooth muscle actin, a marker for fibroblast activation, was not significantly increased following 0.06 µg/ml particle exposure in any treatment group (Fig. 10C). Previous studies have demonstrated the key role of TGF-β1 as an upstream modulator of pulmonary fibrosis. TGF-β1 was also variable between particle exposures and doses in a manner that did not always correlate to dose (Fig. 10D). However, as a general trend, materials MW #4–7 and CNF #1–2 induced greater TGF-β1 production.

### Hierarchical clustering and PCA of the fibroblast outcomes with physicochemical characteristics

**Fig. 11:**
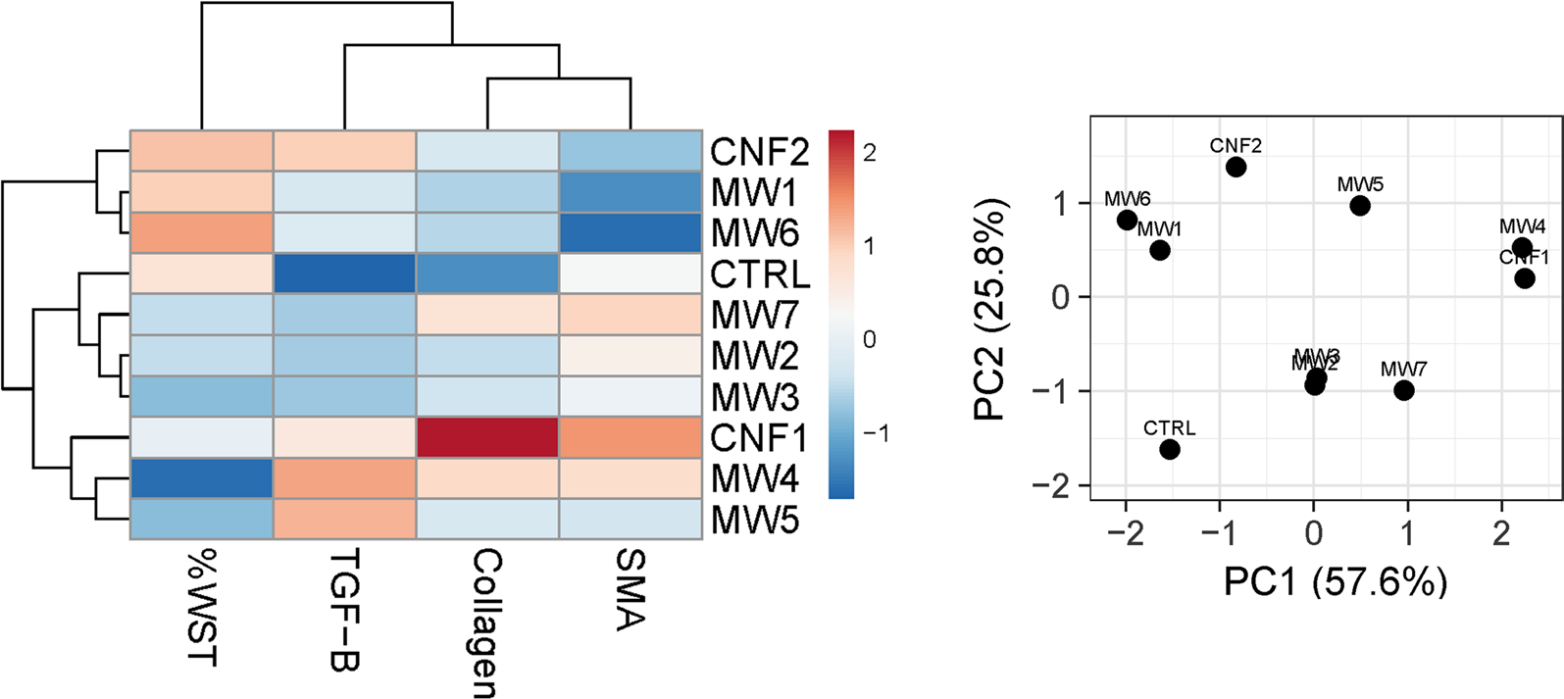
Hierarchical clustering analysis (HCA) and principal component analysis (PCA) comparing in vitro toxicity outcomes of human fibroblast cells treated with CNT/F materials

The initial analysis of the four fibroblast endpoints (Fig. 11), cell viability with collagen, αSMA, and TGFβ1 protein levels, did not correspond to in vivo grouping for all histopathological outcomes (Fig. 6) or separation of alveolar (Fig. 7B) or bronchiolar outcomes (Fig. 7A) especially with MW #7 closely associated with MW #2, #3, and control cells. The addition of the physicochemical characteristics (Additional file 1: Figs. S10 and S11) with outcomes offered something closer to the in vivo outcomes in that MW #7 and CNF #1 grouped as did MW #1 and control cells. While this was the case, collagen and TGFβ1 production grouped separately. Collagen and SMA grouped with larger lengths and diameters while TGFβ1 grouped with smaller length and diameters (Additional file 1: Figs. S10 and S11B). Previous in vitro studies have investigated the role of TGFβ1 in collagen production and found that SWCNT and MWCNT induced TGFβ1 production and subsequent collagen-1 production through the SMAD signaling cascade [44]. Furthermore, TGFβ1 and SMA are associated with the epithelial-mesenchymal transition (EMT) of pulmonary epithelial cells to a migratory cell phenotype associated with cancer and metastasis, and has been found to drive similar EMT outcomes in other nanoparticle exposures including cerium oxide [46–48].

While the in vitro data did not fully represent findings in vivo, we can conclude that fibroblast monocultures alone may not accurately represent in vivo findings due to the complex interactions of several cell types and the structure of the extracellular matrix. Alternative and/or more advanced methods, such as co-culture and air–liquid interface models, along with a broader set of fibrosis markers, may be more inclusive of the complexities absent in monoculture and may be employed in future experiments. Had a smaller in vitro study been conducted, such as MW #1 compared to MW#7 or CNF#1, the conclusion could have been made that larger physical diameter and length materials cause a greater response when considering outcomes alone. When examining a broader set of materials at one time, those conclusions were not apparent.

### Summary

The overall goal of this study was to assess severity in pulmonary histopathology at a sub-chronic time point from CNT/F exposure of various physicochemical characteristics. In depth histopathological analysis was completed to assess both the severity of tissue changes and injury as well as the regional distribution of those changes. The standard toxicological outputs indicated obvious differences between materials that was put into perspective using computational analyses.

Previously with a combination of measured genotoxic responses [20], CNT/F with larger physical dimensions, a combination of length and diameter, conferred greater toxicity. This was generally the case for histopathology outcomes, especially MW #5–7 and CNF #1. Regional specificity was noted for some. For example, MW #2, a highly entangled material, only had bronchiolar effects while CNT #2 lacked marked bronchiolar effects. Distribution and, thus, regional deposited dose may have influenced the specificity of histopathologic effects. The importance of combined length and width was the separation between CNF #2 and MW #5–7 and CNF #1, as CNF #2 had a reduced length and bundled agglomerate mean length. MW #1 and #3, while not observed as singlets in the lung, were not without effect. Those materials, primarily in the L2, L4, and W 0.05 bins exhibited granulomatous inflammation potential because the small spherical bundles can easily deposit in the deep airways. The lack of larger physical diameter nominal tubes and singlets in MW #1 and #3 was the likely reason for a lack of bronchiolar effects and pronounced alveolar fibrosis.

### General conclusions

- The broad class of CNT/F resulted in variable pathology and severity of outcomes. The smaller diameter and length materials, present in most all CNT/F, consistently resulted in granulomatous inflammation and alveolar effects. The addition of populations of CNT/F with both increased nominal tube diameter and increased length induced bronchiolar pathology and increased the severity of alveolar pathology. The results emphasize toxicity associated with the inhalable fraction.
- CNT/F containing nominal tubes of increased length and diameter caused ectasia of the lymphatics. Accumulation at the bronchoalveolar junction may contribute to alveolar fibrosis and pleural accumulation by obstructing clearance mechanisms.
- Similar to our previous evaluations, binning of physical dimensions (length and diameter/width) offered greater resolution when determining specific physicochemical characteristics contributing to various aspect of developing pathology [20]. The combination of how the CNT/F agglomerate, or two-dimensional sizing, and binning of nominal tube physical dimensions were excellent segregators predicting pathology. Traditional characterization of means only did not offer great resolution for effect determination.
- The well-studied benchmark material, MW #5/Mitsui-7/MWCNT-7, was not distinct in comparative effect. Some CNT/F had similar pathological characteristics while other CNT/F did not exhibit the toxicity profile following pulmonary exposure. Comparative toxicity of the various CNT/F can aid risk assessment.
- In terms of toxicity outcome alone and our study design, human fibroblast cells did not group materials as did the in vivo evaluations. Perhaps a different approach, other than a single cell submerged cell culture, would provide more compatible outcomes.
- As previously indicated for genotoxicity outcomes [20], the increased length and diameter CNT/F contributing to greater severity in pathological outcomes do not need to be the major fraction of nominal tubes in the sample. A small proportion of tubes with those characteristics were sufficient to alter the severity of the toxicity profile.

## Materials and methods

### Materials

Nine materials were assessed in this study including seven multi-walled carbon nanotubes and two carbon nanofibers and are the same materials used in this series of studies. These materials were produced or used in U. S. facilities except for MW #5, the well-known MWCNT-7/Mitsui-7 which was used as a benchmark material in this study. These materials were extensively characterized as previously reported and characteristics are provided in Additional file 1: Tables S1–S3 and Additional file 1: Figs. S1 and S2 [20].

### In vivo study design

Male C57BL/6 J mice aged eight to ten weeks were exposed by oropharyngeal aspiration to either vehicle (physiologic dosing medium; DM), one of seven multi-walled carbon nanotubes (MW #1–#7), or one of two carbon nanofibers (CNF #1–#2). Only the high dose of 40 µg was used in this study. Previous studies have shown that a 40 µg dose of the benchmark material was necessary to induce pathological changes, making it necessary for this study [3, 6, 22]. Mice were euthanized at 84 d post-exposure and tissue was collected to assess changes in histopathology.

### Animals

Male C57BL/6 J pathogen-free mice weighing 20–25 g were obtained from Jackson Laboratories (Bar Harbor, ME) and were housed in the AAALAC International-accredited NIOSH animal facility. Mice were provided food and tap water ad libitum in ventilated cages, on autoclaved hardwood chip bedding and an environment of controlled humidity, temperature, and 12:12 light/dark cycles. Animals were allowed to acclimate for at least seven days prior to use in any experiments. Animal care and use procedures were conducted in accordance with the “PHS Policy on Humane Care and Use of Laboratory Animals” and the “Guide for the Care and Use of Laboratory Animals” (2011) and the procedures utilized in this study were approved by the Centers for Disease Control and Prevention, Morgantown Institutional Animal Care and Use Committee.

### Facility representative material preparation and in vivo dosing

Fresh physiologic dosing medium (DM) was prepared prior to dosing. DM contained mouse serum albumin (0.6 mg/ml) and 1,2-dipalmitoyl-sn-glycero-3-phosphocholine (DPPC; 0.01 mg/ml) prepared in United States Pharmacopeia (USP) grade-phosphate buffered saline (PBS) without calcium and magnesium. To disperse the particles in DM, samples were sonicated for 5 min at the highest setting using an external sonicator (Hielscher Ultrasound Technology) and then for 5 min using a Branson Sonifier 450 probe sonicator set to the lowest setting (10% duty cycle; output control of 1). Mice were dosed by oropharyngeal aspiration according to the well-established protocols previously described [49].

### Tissue collection

Mice were sacrificed at 84 d post-exposure. Following an intraperitoneal injection of 100–300 mg/kg sodium pentobarbital, the abdomen was exposed, and mice were exsanguinated. The right lung was inflated with 10% buffered formalin by gravity fixation. Fixed tissue was then embedded in paraffin, cut into 5 µm sections, and mounted on slides for staining.

### Immunofluorescent staining

Paraffin embedded lung was sectioned and mounted on ProbeOn Plus slides (Thermo Fisher Scientific, Pittsburgh, PA). Sections were deparaffinized by heating in a 60 °C oven for 20 min and then immersing in three sequential xylene baths and rehydrated in descending alcohol solutions followed by distilled water. Antigen retrieval was achieved by placing slides in 1 mM ethylenediamine tetraacetic acid (EDTA), pH 8.0 and brought to boil via microwave. Non-specific staining was blocked with 10% donkey serum (Jackson Immuno Research Laboratories 017–000-121, West Grove, PA) in PBS. The primary antibodies, a 1:100 dilution of podoplanin (Novus Biologicals NB600-1015, Littleton, CO) in PBS and a 1:50 dilution of E-cadherin (BD Biosciences BD 610,181, San Jose, CA) in PBS, were applied via capillary action and incubated with the slides overnight at 4 °C. Non-specific binding was blocked with 10% donkey serum in PBS. DyLight 594 AffiniPure goat anti-syrian hamster IgG (Jackson Immuno Research Laboratories 107-515-142, West Grove, PA) and Alexa Fluor 488 AffiniPure donkey anti-mouse IgG (Jackson Immuno Research Laboratories 715-545-150, West Grove, PA) were the secondary antibodies.

### General histopathology

Lung tissue from 84 d post-exposure was sectioned, mounted on slides, and stained with either Masson’s Trichrome stain or hematoxylin and eosin (H&E). The right lung of the mouse was embedded and sectioned. The right lung of the mouse has four lobes to offer evaluation from multiple lobes. Histopathology was evaluated by a board-certified veterinary pathologist and the semi-quantitative scores included assessment of distribution and severity as recommended for standard toxicologic pathology evaluation [50]. Two semi-quantitative scores were assigned for each morphologic alteration. The severity score is the numerical equivalent of the following intensities of tissue morphologic change: 0 = none, 1 = minimal, 2 = mild, 3 = moderate, 4 = marked, and 5 = severe. The second was a distribution score to quantify the extent (amount) of the tissue involvement. The distribution scoring is as follows: 0 = none, 1 = focal (single small area), 2 = locally extensive (single larger area), 3 = multifocal (many small areas), 4 = multifocal and coalescent (many small areas with some merging), and 5 = diffuse (widespread involvement). These scores were combined to generate a total score ranging from 0 to 10 as previously described [51]. Photomicrographs were captured using an Olympus BX53 microscope equipped with a DP73 camera (Olympus, Tokyo, Japan).

### Morphometry and alveolar fibrosis

Morphometric analysis was used to measure changes in alveolar fibrosis. Lung sections from 84 d post-exposure were stained with picrosirius red to detect collagen fibers as previously described [22]. Briefly, 5 µm sections of lung were immersed in 0.1% Picrosirius red solution for 1–2 h and were rinsed with 0.01 N HCL. Subsequent counterstaining with hematoxylin was completed followed by coverslipping. Quantitative morphometric analysis was used to determine the volume density of alveolar collagen and measure alveolar collagen thickness using basic point and intercept counting. Point and intercept counts were made using an 11-line overlay graticule (12.5 mm square with 100 divisions), at 100× magnification, taken at eight locations equally spaced across each section. One section was used per animal with a total of 4–7 animals per treatment group.

### Bronchiolar fibrosis

To assess the severity of bronchiolar fibrosis at 84 d post-exposure in lung sections stained with Masson’s Trichrome stain, images of the broncho-alveolar duct junction in the most affected regions were taken using an Olympus BX63 microscope equipped with a DP73 camera and CellSens Dimension software (Olympus Corporation, Tokyo, Japan). For each lung section, a total of six images were taken. Three images were taken of junctions with minimal severity of bronchiolar fibrosis. Additionally, three images were taken of most affected junctions representing the most severe fibrosis. Only three images were taken of control animals as there were no “affected” regions. For each treatment, one section per mouse was taken for a total of n = 4–7 per treatment group. Bronchiolar fibrosis was quantified using ImageJ (National Institutes of Health, Bethesda, MD) as previously described [52]. Briefly, using the color deconvolution plugin, the image was separated into color channels, with green representing areas of fibrosis. The areas of bronchiolar fibrosis were selected and quantified. The terminal bronchiole at the bronchioloalveolar duct junction was selected and the region of interstitial tissue extending from the basement membrane of the terminal bronchiole to the alveoli was outlined. No lumen was measured. The area was divided by the total area of the field of view to quantify the total percent area of fibrosis. Additionally, the area was then normalized to the length of the basement membrane length of the terminal bronchiole to express the fibrosis as a measurement of area per mm of basement membrane.

### In vitro study design

Primary normal human lung fibroblasts (NHLFs) were exposed to either DM or one of the nine CNT/F at doses relevant to human occupational exposure to assess cytotoxicity and the potential mechanisms by which histopathological changes may be occurring. Histopathological changes in the tissue involves integrated action of multiple factors from various cell types. This study aimed to determine the degree to which single cell in vitro assays can be used to predict the complex histopathological in vivo outcomes.

### Cell culture

NHLFs were acquired from Lonza (Walkersville, MD) and cultured at sub-confluent densities in fibroblast growth medium which contained fibroblast basal medium with FGM2 BulletKit growth supplements (FGM2, Lonza) and 100 U/ml penicillin/streptomycin as previously described [53]. Briefly, cells were passaged by washing with a HEPES-based saline buffer, suspension via 0.025% trypsin/EDTA, and neutralization using trypsin neutralization solution (Lonza) following manufacturer’s procedures. Cells were maintained in a 37 °C incubator in a humid, 5% CO_2_ atmosphere. 3rd–7th passage NHLFs were used for all assays.

### CNT/F dispersion in cell culture media

Dry CNT/F were weighed and placed into labeled, separate Eppendorf tubes. Stock suspensions of the 9 CNT/F’s were prepared in well characterized dispersion media (DM) at 3 mg/ml. DM contains 0.6 mg/ml albumin and 10 µg/ml DPPC in sterile phosphate-buffered saline (PBS) and was freshly prepared for each assay. CNT/F were sonicated for 5 min using an external sonicator (Hielscher Ultrasound Technology) at 4 °C as an initial dispersion procedure. Then, each CNT/F was then hand sonicated for 20 s using a microtip sonicator (Fisher Scientific) at 4 °C three separate times and immediately serially diluted in FGM2 medium for cellular assays.

### WST-1 assay

To screen for CNT/F cytotoxicity and changes in cell metabolism, WST-1 assay was conducted as previously described with modifications [43]. Briefly, NHLFs were passaged and seeded into tissue culture-treated 96-well plates (Corning Inc., Corning, NY) at 5,000 cells per well overnight in 100 µl volume. Cells were then exposed to seven dilutions of each CNT/F in fresh medium ranging 0.02–20 µg/cm^2^ in 200 µl volume along with unexposed cells and medium-only blanks for 24 h. All treatment groups were run in triplicate. 1 h prior to the assay time point, 1% Triton-X solution was added to a subset of unexposed cells to serve as a 100% cytotoxicity control. Next, WST-1 reagent was added to each well and the plate was allowed to incubate for 2 h in the culture incubator following manufacturer’s instructions (Roche LifeScience, Indianapolis, IN). Absorbance of metabolized WST-1 was read on a SpectraMax 250 microplate spectrophotometer (Molecular Devices, Sunnyvale, CA) at 462 nm while 650 nm served as a background measure. Absorbance values were subtracted from mean blank values and means calculated. Three independent assays were run. Since high CNT/F concentrations in the wells increased baseline absorbance values, two separate assays with the same CNT/F dose scheme, but without cells, served as particle controls and were used to correct absorbance values for all cellular assays. Mean absorbance values were converted to percent viability.

### Collagen I, α-SMA, and TGFβ1 expression

To provide evidence of direct fibroblast stimulation ability, CNT/F exposed NHLFs were screened for pro-fibrotic markers as previously described [44, 45]. Briefly, suspended NHLFs were seeded into tissue culture-treated 6-well plates (Corning) at 3E5 cells per well overnight. NHLFs were then exposed to 0.02, 0.06, and 0.2 µg/cm^2^ in 2 ml volume for 48 h, which match a 10 µg, 30 µg, and 100 µg dose per mouse lung assuming 500 cm^2^ surface area [45]. Unexposed cells and 1 ng/ml human TGFβ1 served as negative and positive controls. Since the design was spread across four plates, one well of unexposed cells on each plate served as a plate control. Following exposure, digital phase contrast images of dispersed CNT/F on NHLFs were acquired at 10X and 20X using a Revolve microscope (ECHO, San Diego, CA). Cell lysates were prepared for western blot analysis. Briefly, after plates were chilled on ice for 5 min, conditioned medium was collected into Eppendorf tubes, centrifuged at 1000 rpm for 5 min to pellet cell debris, followed by the collection and storage of the supernatant at − 80 °C for TGFβ1 assay. Collected conditioned medium was assessed in technical triplicate for secreted total TGFβ1 concentrations via DuoSet ELISA after latent TGFβ1 activation via acid incubation following manufacturer’s instructions (R & D Biosystems, Minneapolis, MN) as previously described [44]. Absorbance was measured at 450 nm on a 96-well microplate reader.

Next, exposed NHLFs were washed in cold PBS, followed by incubation in lysis buffer (Invitrogen, Carlsbad, CA) containing 0.1 mM PMSF and complete protease inhibitor cocktail. Lysate samples were scraped, collected into tubes, briefly homogenized using microtip sonication, and centrifuged at 12,500 rpms for 15 min at 4 °C. Collected supernatants were assayed for total protein using a BCA kit following manufacturer instructions (Pierce, Rockford, IL). 30 µg protein samples were separated on 7% SDS-PAGE gels followed by semi-dry transfer (Fisher Scientific, Hampton, NH) to nitrocellulose membranes. Following blocking in 0.5% or 5% non-fat dry milk in Tris-buffered saline with 1% Tween20 (TBS-T), membranes were probed for rabbit Collagen I (Fitzgerald, Acton, MA), rabbit α-SMA (AbCam, Cambridge, MA), and mouse monoclonal β-actin (Sigma Aldrich) primary antibodies using either MiniBlot 2.0 system at room temperature (Millipore, Burlington, MA) or overnight at 4 °C. After rinsing thrice with TBS-T, membranes were incubated with either rabbit or mouse HRP-conjugated secondary antibodies (Santa Cruz Biotechnology, Dallas, TX) for 1 h at room temperature. Lastly, membranes were incubated with SuperSignal chemiluminescent substrate (ThermoFisher Scientific, Waltham, MA) for 5 min and then exposed to X-ray film. Films with bands were digitized and densitometry performed on ImageJ. Protein expression was calculated as fold change compared to unexposed controls following correction using β-actin expression for each sample. All experiments were performed three independent times.

### Statistics

Data are presented as mean with standard error or standard deviation as indicated in the figure legends. Figures were prepared using SigmaPlot software (Systat Software, INC). Statistical analysis used include one-way analysis of variance (ANOVA), and two-way ANOVA (Dose by Treatment). Histopathological scoring were analyzed using Kruskal–Wallis tests and pairwise post-hoc comparisons were generated using Wilcoxon Rank-Sum tests. Differences were considered significant at *p* < 0.05. This analysis was performed using SAS/STAT software, Version 9.4 of the SAS system for Windows, and JMP version 12.2 (SAS Institute, Cary, NC).

### Principal component analysis and hierarchical clustering analysis

Using traditional, L-W, and combined variables, principal component analysis (PCA) was performed to identify significant patterns that explained the majority of the variations in the pathological responses and physicochemical properties among the different CNT/F materials investigated. PCA was performed using the prcomp command of the R statistical software (R Core Team, 2016).

To further identify groups of CNT/F materials with similar pathological responses, hierarchical cluster analysis was performed. Unit variance row scaling method was employed to normalize the values. Hierarchical agglomerative (bottom up) clustering analysis using R was applied to group pathological responses and the different CNT/F materials employed. The analysis was performed using “Euclidean” distance similarity between different samples and by employing ward.D2 linkage distance between the members of the clusters [54–56]. By combining the clustering of physicochemical characteristics and pathological responses, heat maps were created with colors corresponding to the relative levels of the changes in responses. The heat map and clusters, with similar pathological response profiles and traditional/L-W characteristics corresponding to various CNT/F materials investigated, were produced using package heatmap built for R version 3.1.3 (R Development Core Team2010).

## Supplementary Information

**Additional file 1**. Supplementary tables and figures.

## Data Availability

All data from this project will be posted in the NIOSH Data and Statistics Gateway https://www.cdc.gov/niosh/data/default.html.
