# Supplementary material for: Histopathology of the broad class of carbon nanotubes and nanofibers used or produced in U.S. facilities in a murine model
Source: Part Fibre Toxicol. 2021 Dec 20;18:47. doi: 10.1186/s12989-021-00440-z (PMC8686255; doi:10.1186/s12989-021-00440-z)

**Additional file 1**

**Histopathology of the broad class of carbon nanotubes and nanofibers used or produced in U.S. facilities in a murine model**

Kelly Fraser^1,2^ ([yfd3@cdc.gov](mailto:yfd3@cdc.gov)), Ann Hubbs^1^ ([afh0@cdc.gov](mailto:afh0@cdc.gov)), Naveena Yanamala^3^ ([yanamala.naveena@rutgers.edu](mailto:wqu1@cdc.gov)), Robert R. Mercer^1^ ([rpm7@cdc.gov](mailto:rpm7@cdc.gov)), Todd A. Stueckle^1,2^ ([jux5@cdc.gov](mailto:jux5@cdc.gov)), Jake Jensen^1^ ([jjensen@g.harvard.edu](mailto:jjensen@g.harvard.edu)), Tracy Eye^1^ ([tmh7@cdc.gov](mailto:tmh7@cdc.gov)), Lori Battelli^1^ ([lob0@cdc.gov](mailto:lob0@cdc.gov)), Sidney Clingerman^1^ ([okp5@cdc.gov](mailto:okp5@cdc.gov)), Kara Fluharty^1^ ([knk0@cdc.gov](mailto:knk0@cdc.gov)), Tiana Dodd^1^ ([mty2@cdc.gov](mailto:mty2@cdc.gov)), Gary Casuccio^4^ ([gcasuccio@rjleegroup.com](mailto:gcasuccio@rjleegroup.com)), Kristin Bunker^4^ ([kbunker@rjleegroup.com](mailto:kbunker@rjleegroup.com)), Traci L. Lersch^4^ ([tlersch@rjleegroup.com](mailto:tlersch@rjleegroup.com)), Michael L. Kashon^1^ ([mqk1@cdc.gov](mailto:mqk1@cdc.gov)), Marlene Orandle^1^ ([yjj6@cdc.gov](mailto:yjj6@cdc.gov)), Matthew Dahm^5^ ([iwa6@cdc.gov)†](mailto:iwa6@cdc.gov)†), Mary K. Schubauer-Berigan^5,6^ ([BeriganM@iarc.fr)](mailto:BeriganM@iarc.fr)†), Vamsi Kodali^1,2^ ([ywu0@cdc.gov](mailto:ywu0@cdc.gov)), Aaron Erdely^1,2^ ([efi4@cdc.gov](mailto:efi4@cdc.gov))

^1^Health Effect Laboratory Division, National Institute for Occupational Safety and Health, Morgantown, WV; ^2^West Virginia University, Morgantown, WV; ^3^Division of Cardiovascular Disease and Hypertension, Rutgers Robert Wood Johnson Medical School, New Brunswick, NJ; ^4^ RJ Lee Group, Monroeville, PA; ^5^Division of Field Studies Evaluation, National Institute for Occupational Safety and Health, Cincinnati, OH; ^6^International Agency for Research on Cancer, Lyon, France

“The findings and conclusions in this report are those of the authors and do not necessarily represent the official position of the Centers for Disease Control and Prevention, National Institute for Occupational Safety and Health. Where authors are identified as personnel of the International Agency for Research on Cancer / World Health Organization, the authors alone are responsible for the views expressed in this article and they do not necessarily represent the decisions, policy or views of the International Agency for Research on Cancer / World Health Organization.”

**Corresponding author:** Aaron Erdely, PhD

NIOSH/HELD/PPRB

1095 Willowdale Rd, MS-2015

Morgantown, WV 26505-2888

Tel: 304-285-5903

Fax: 304-285-5708

e-mail: [efi4@cdc.gov](mailto:efi4@cdc.gov)


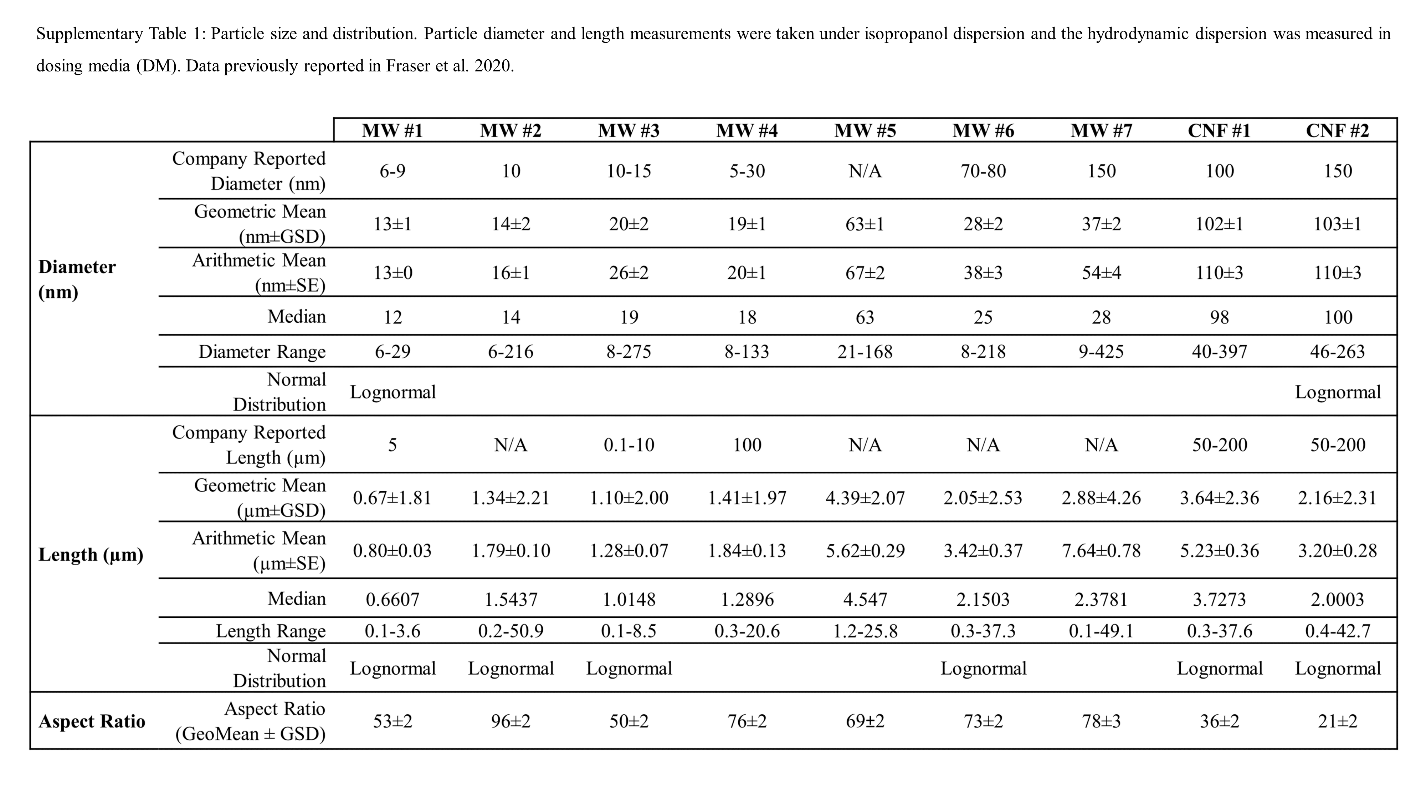

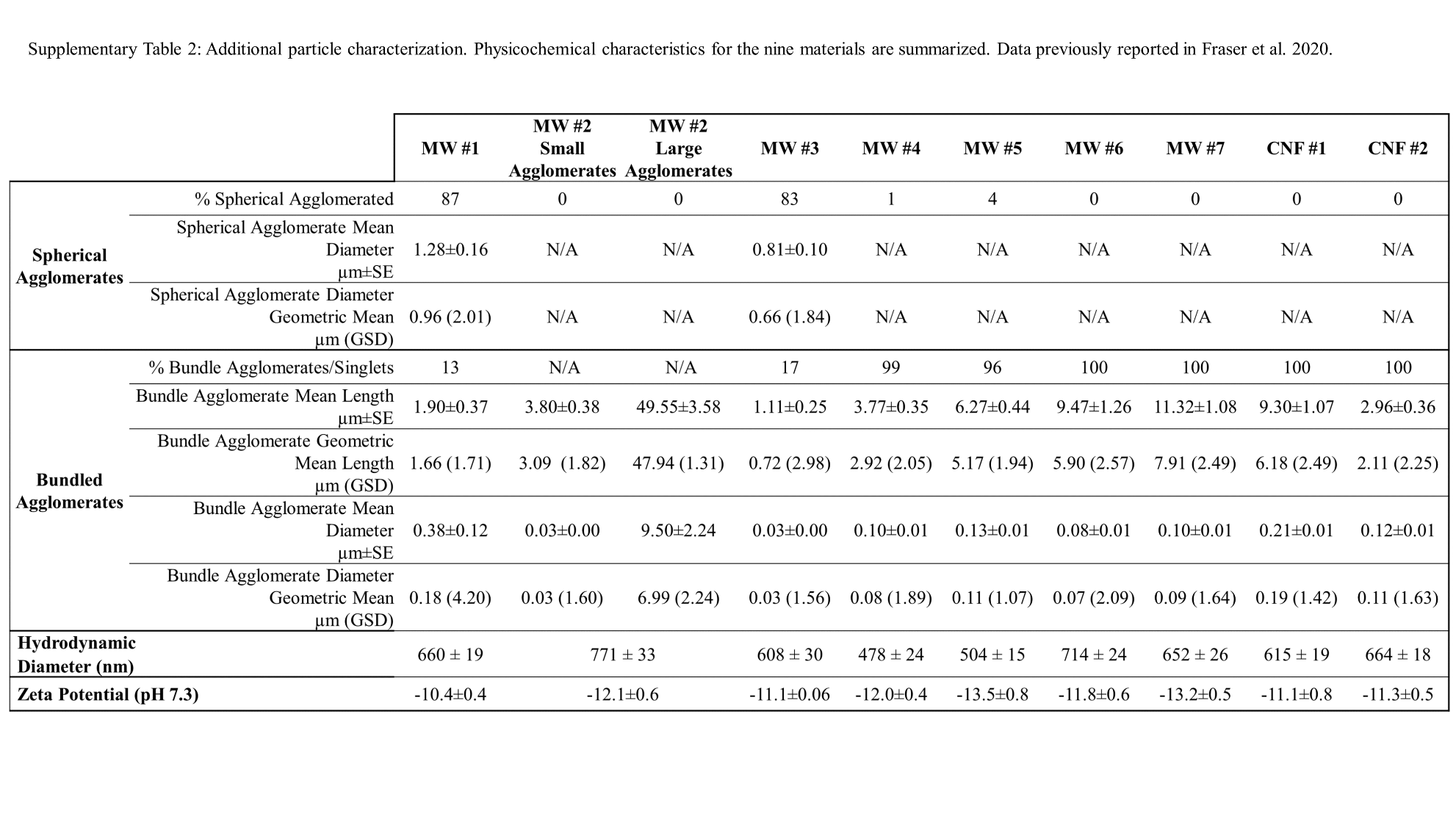

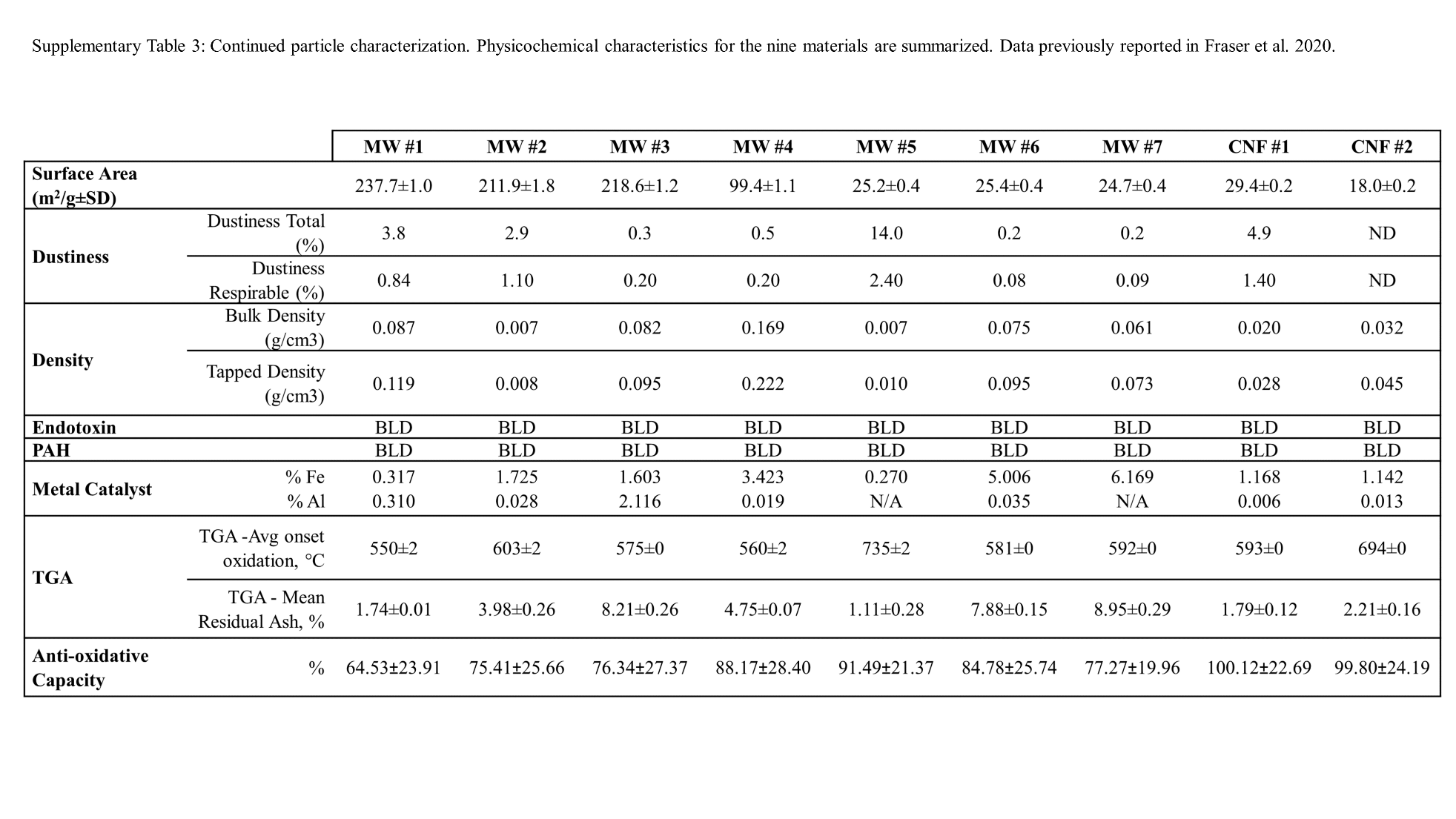


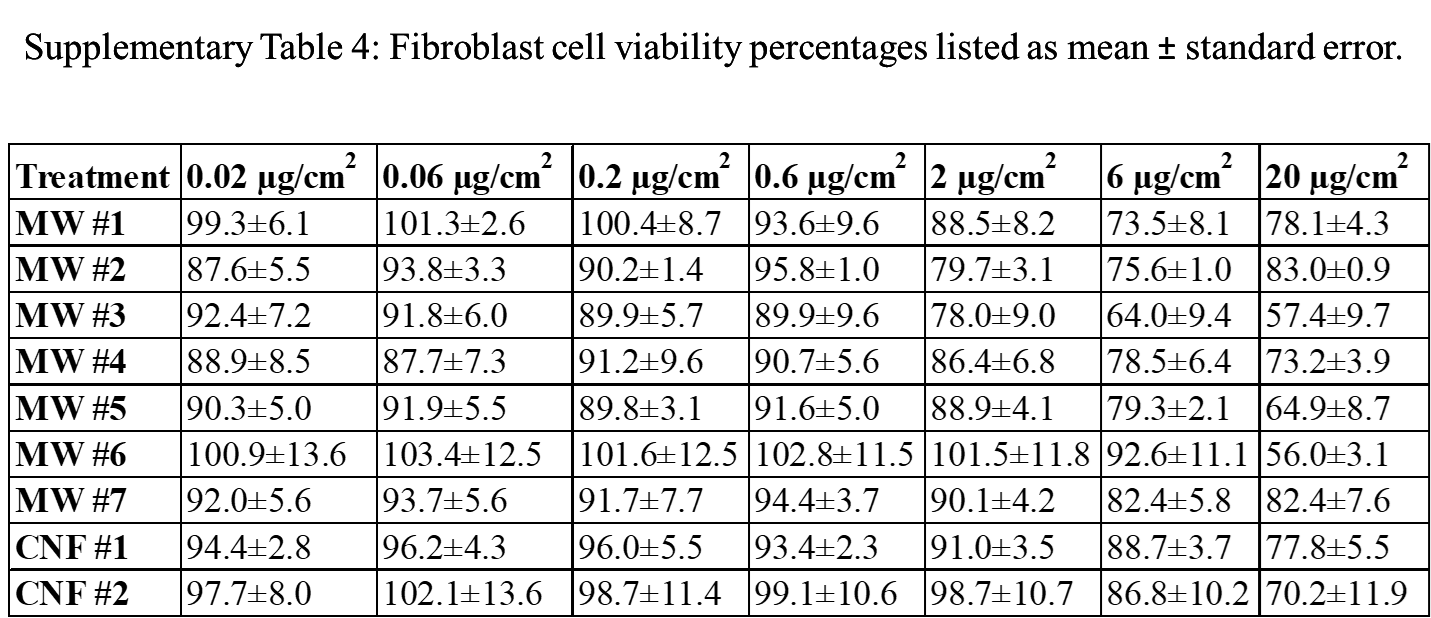


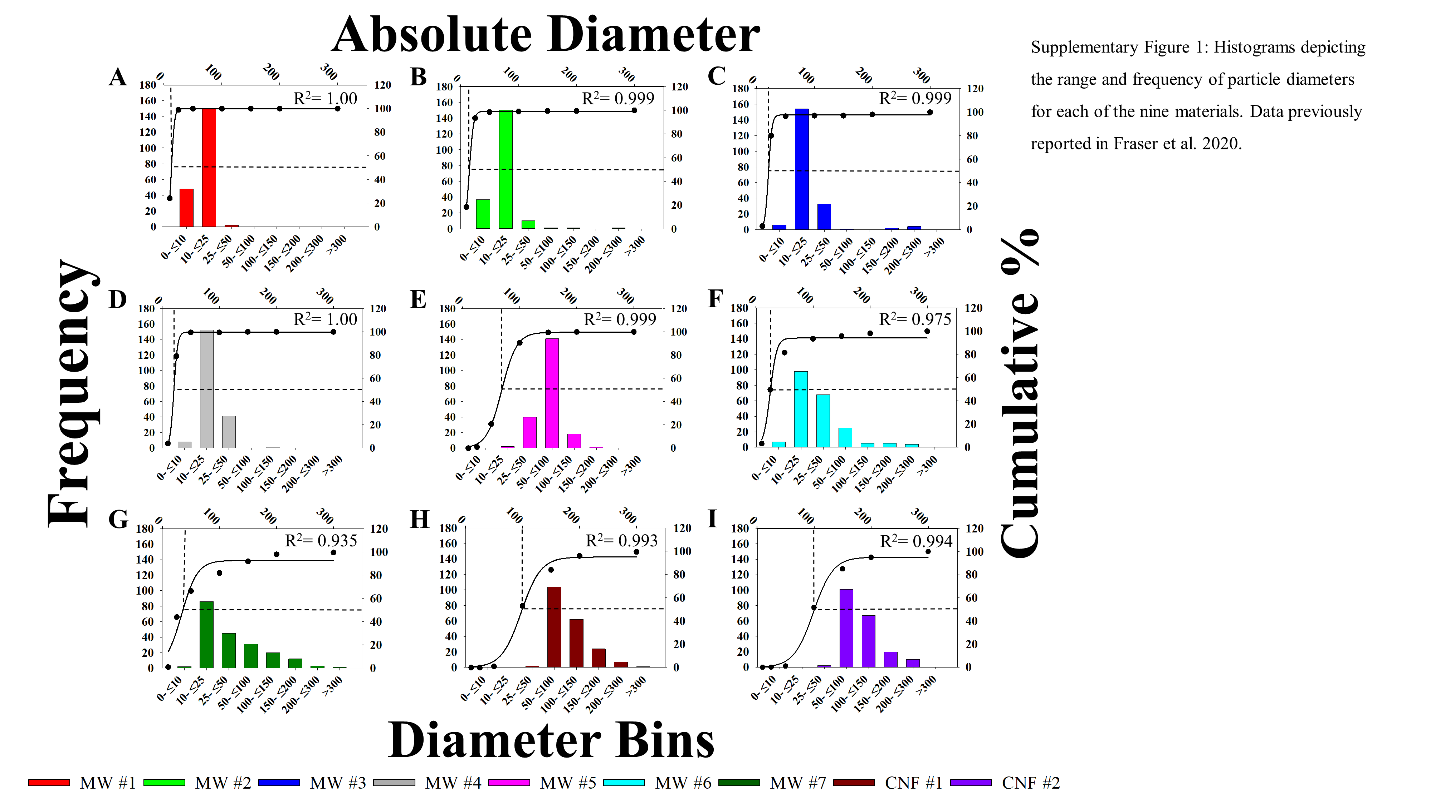


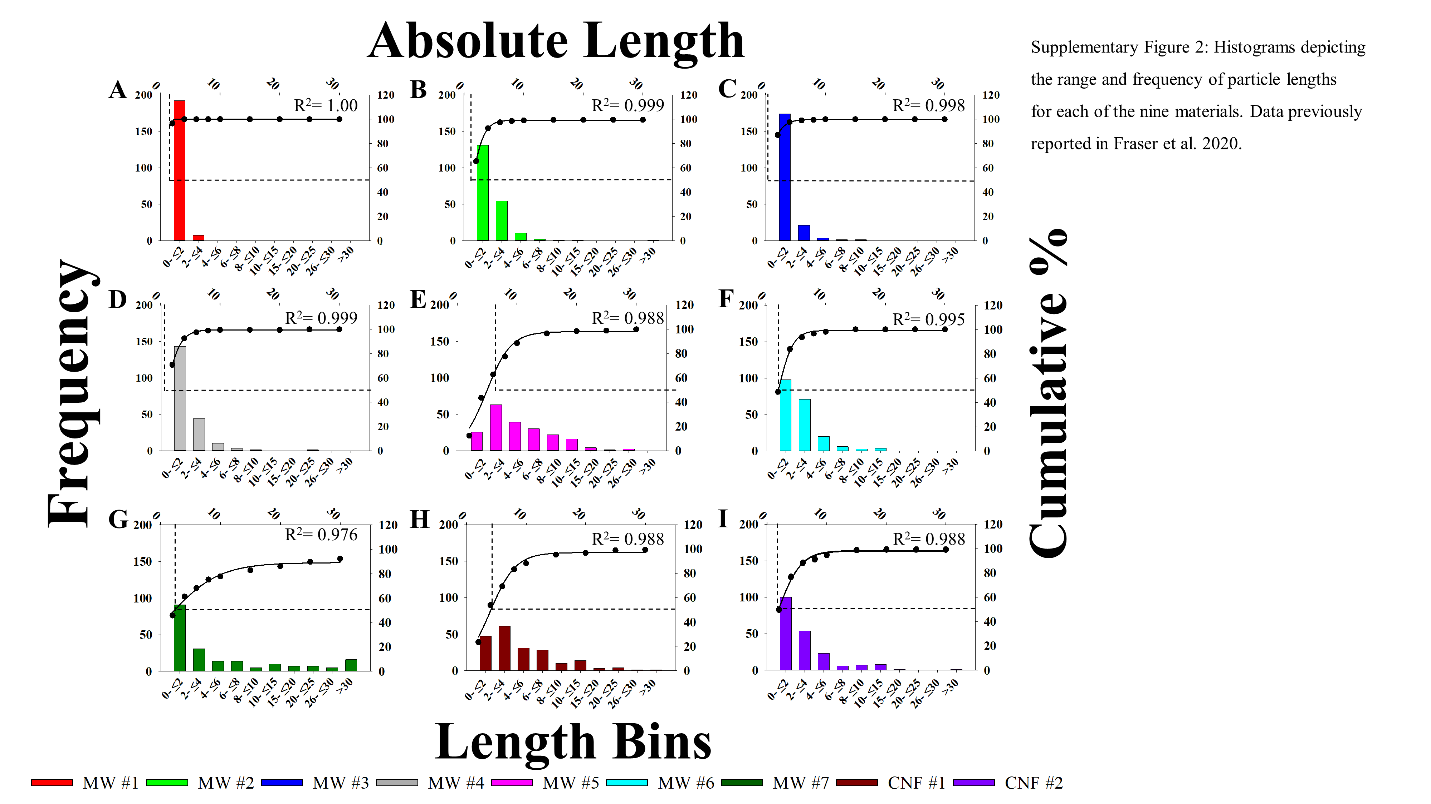


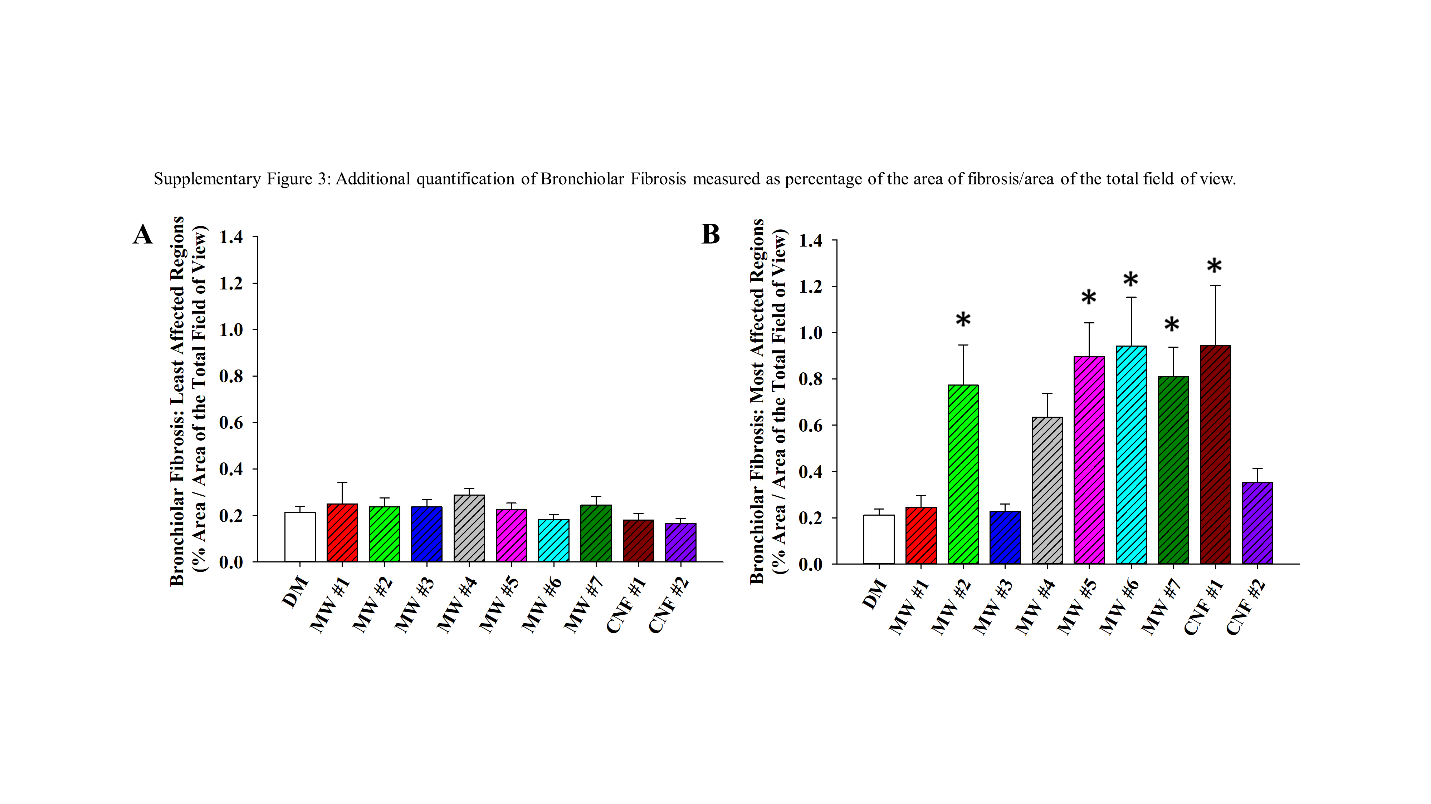


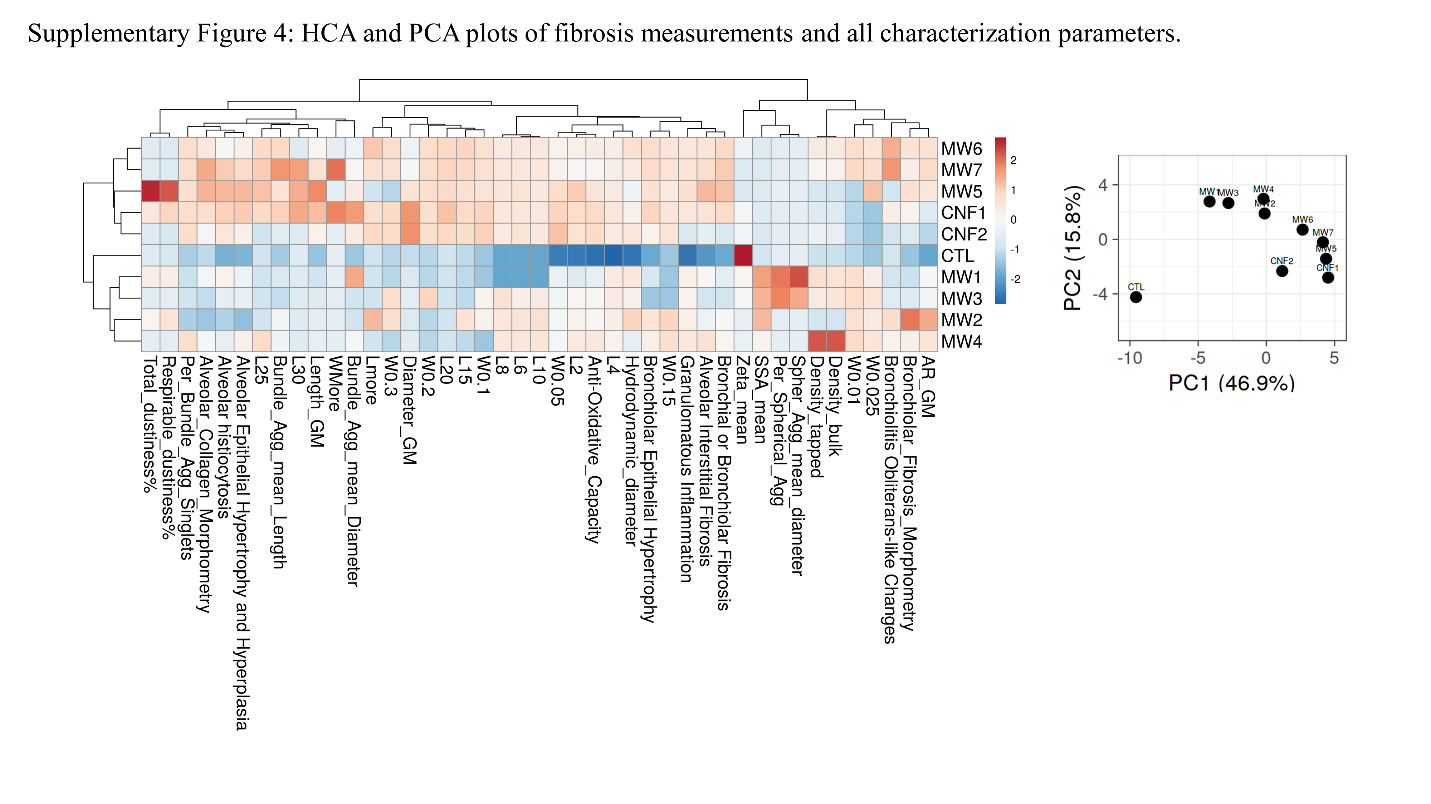


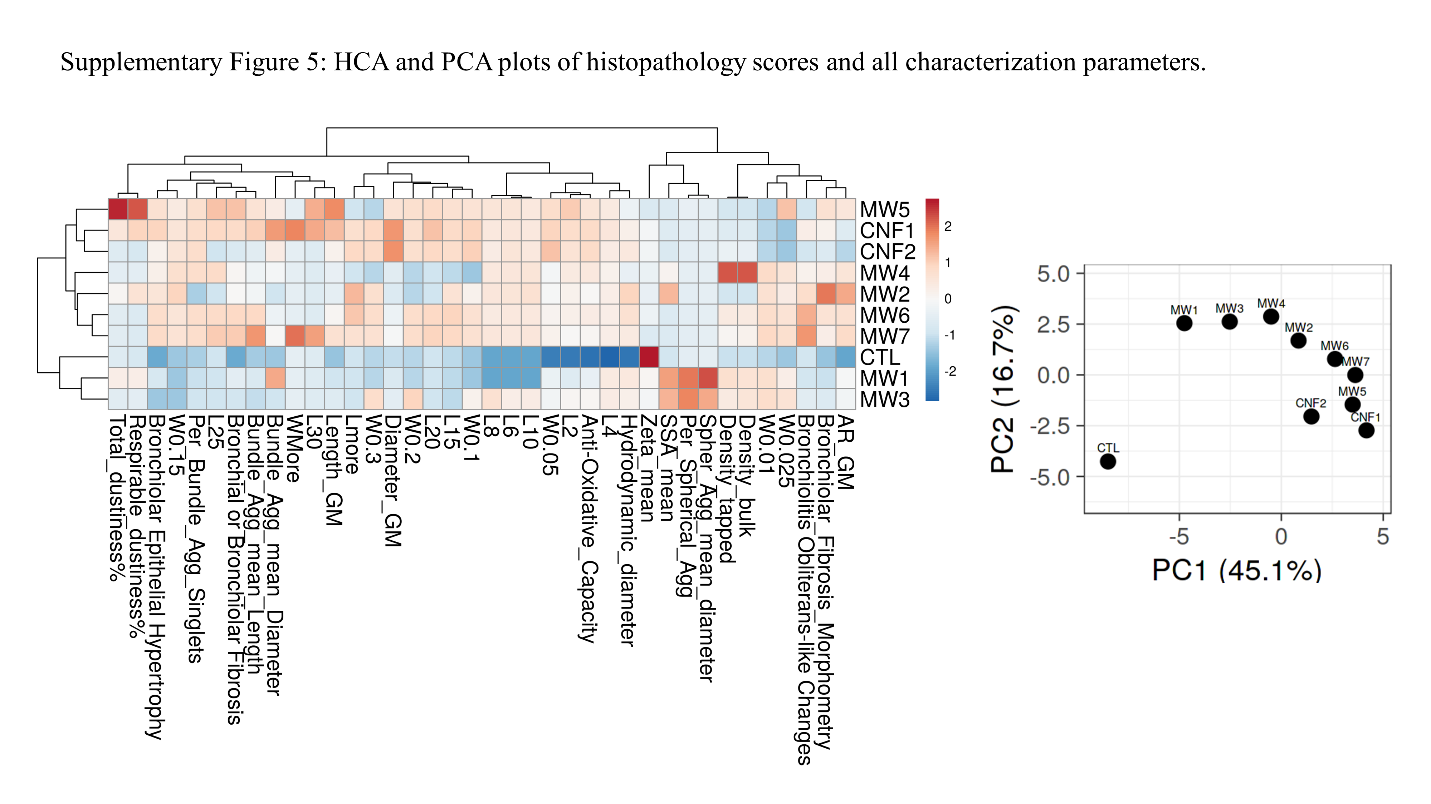


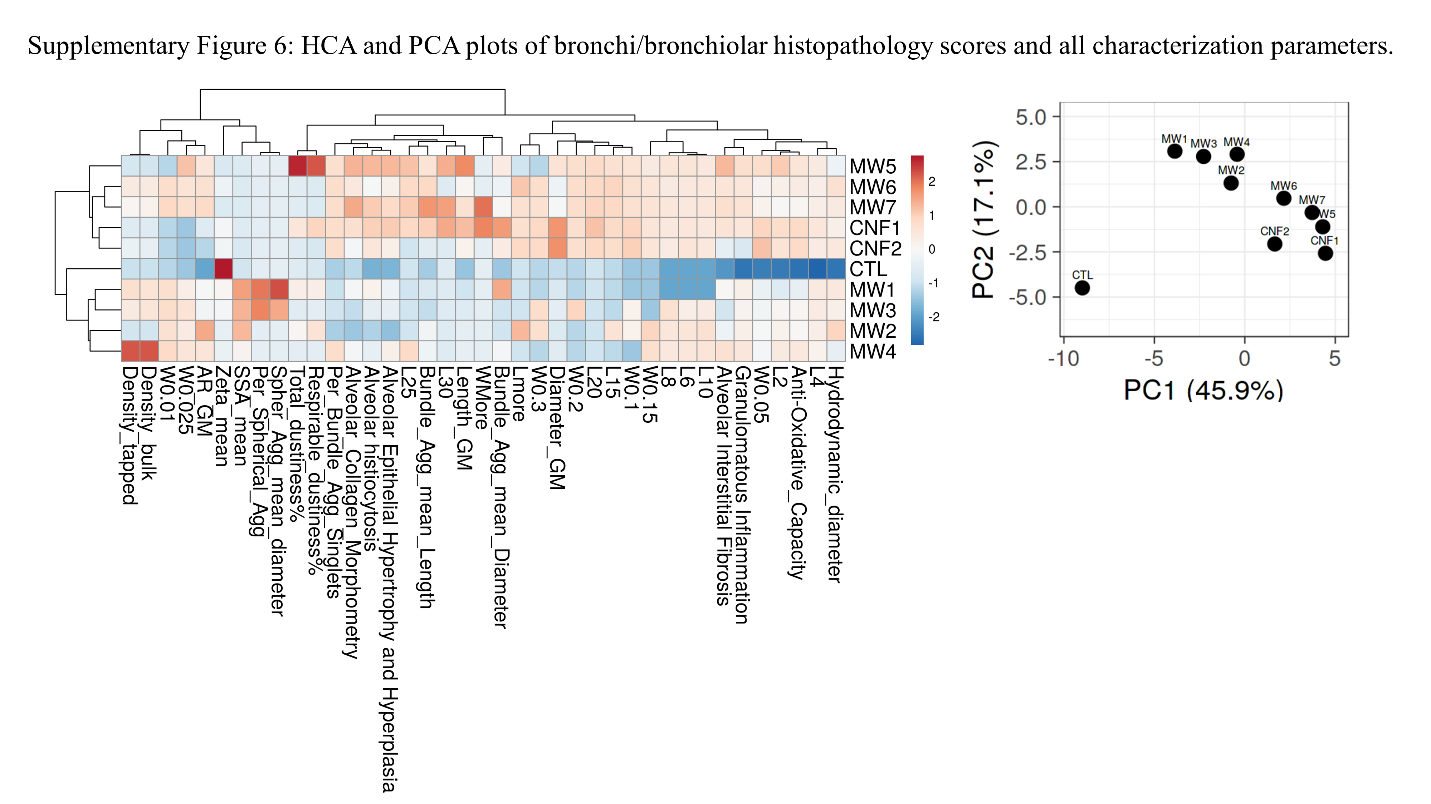


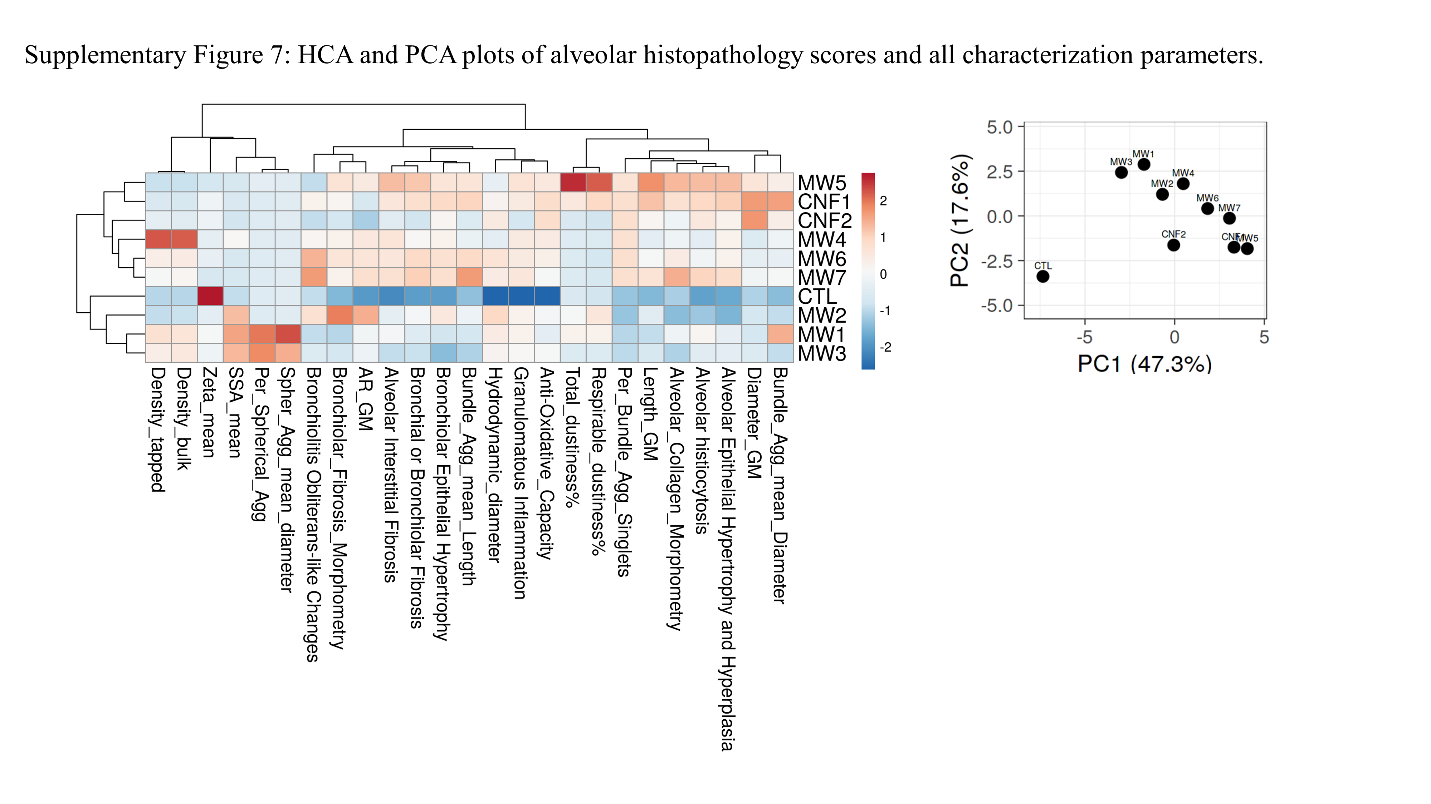


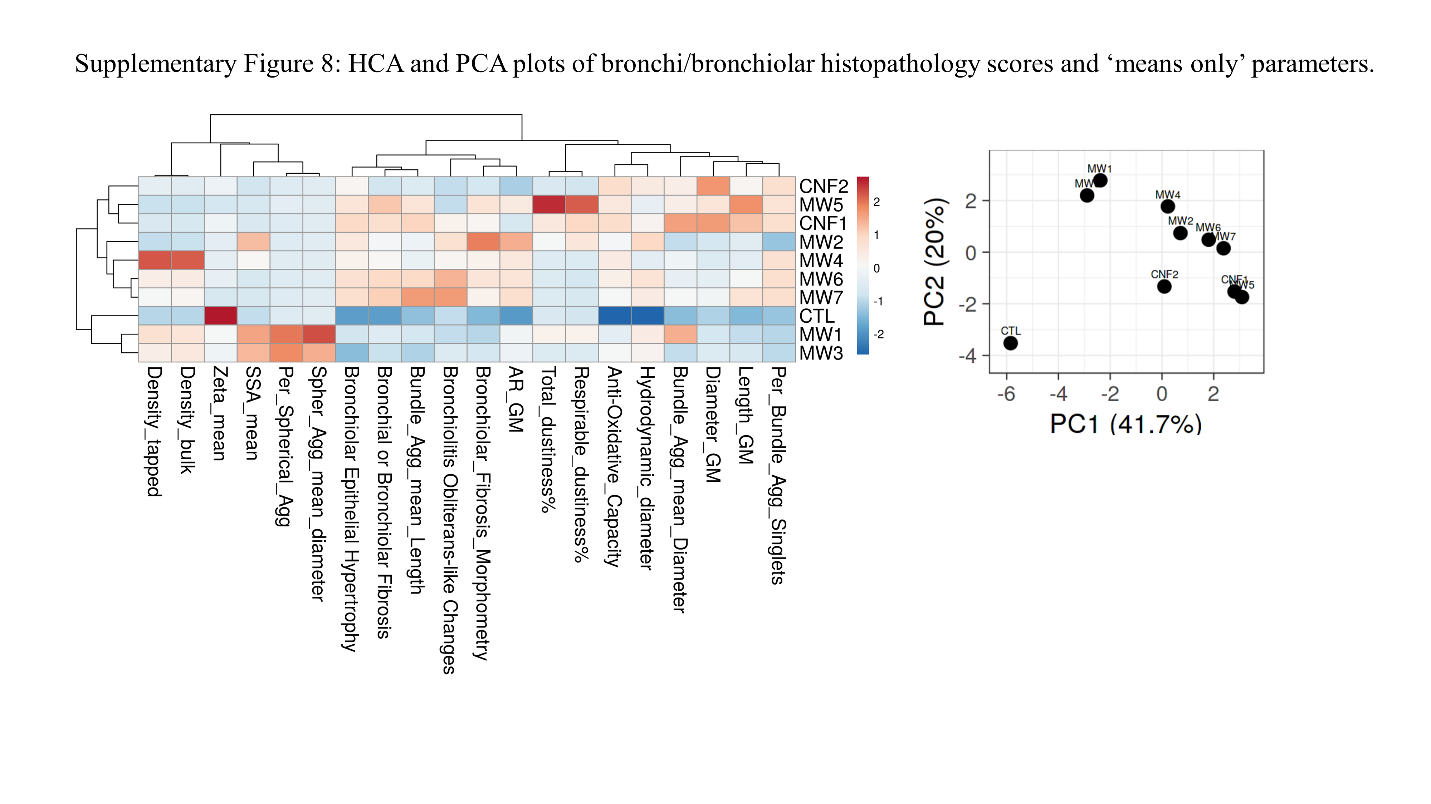


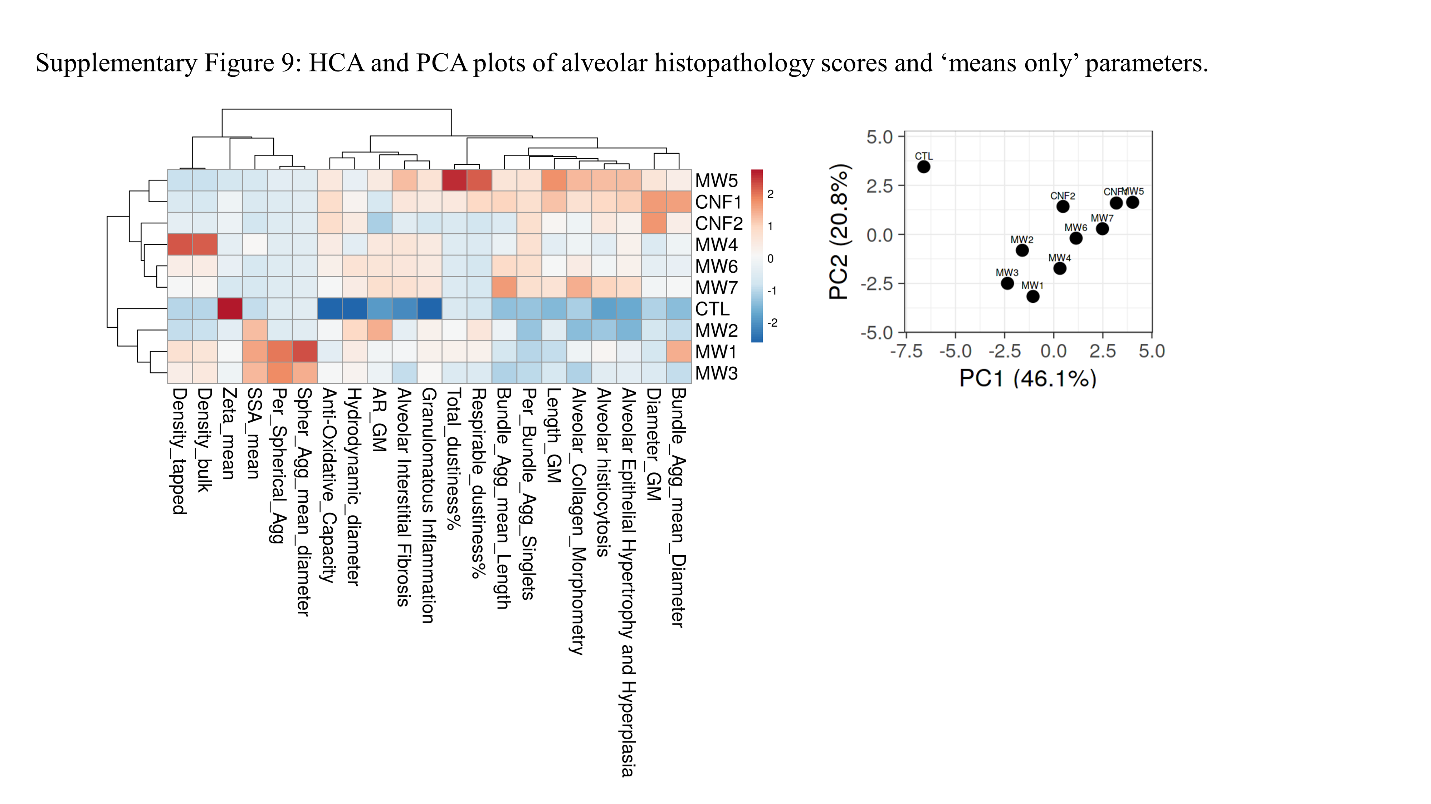


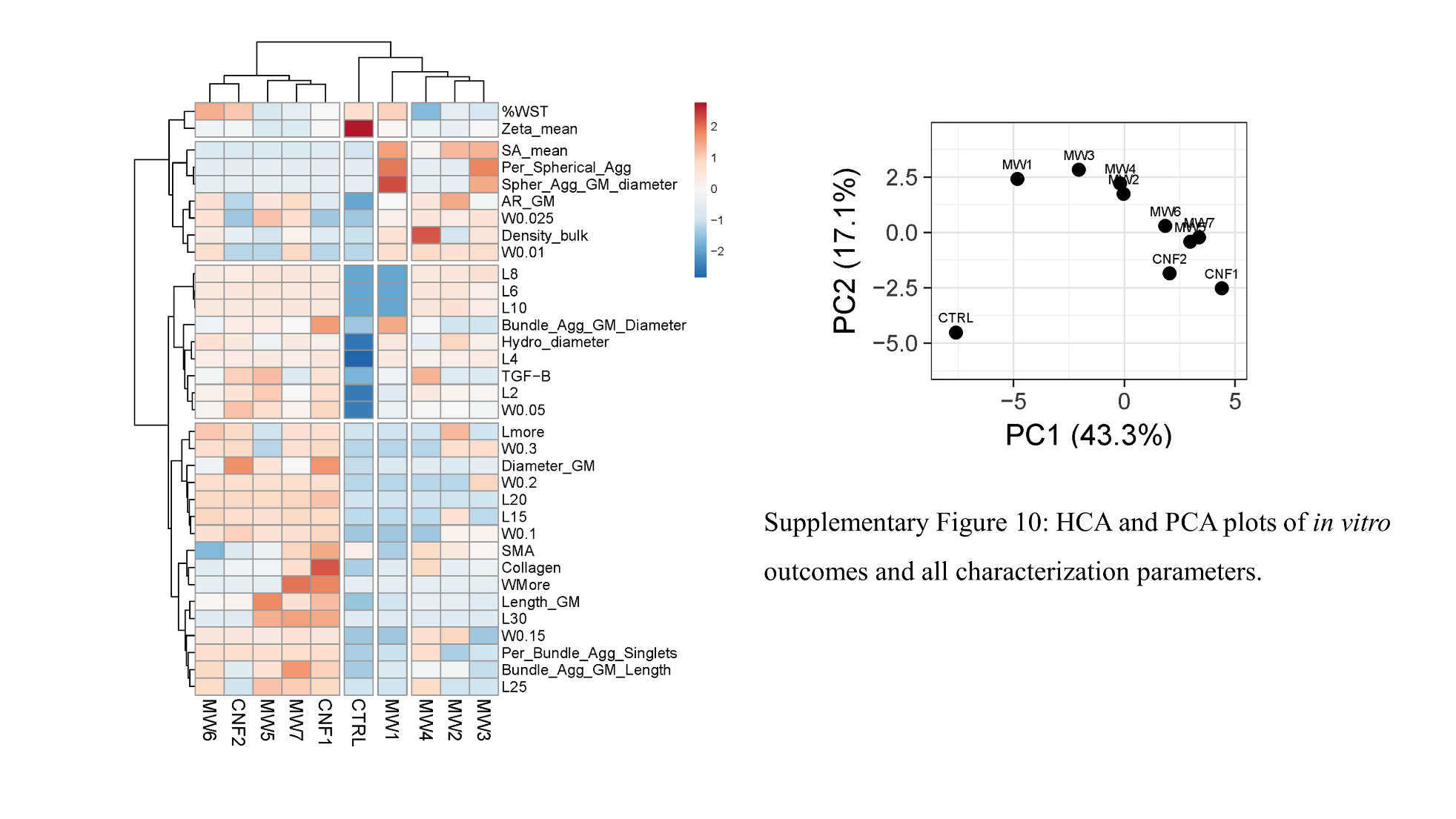


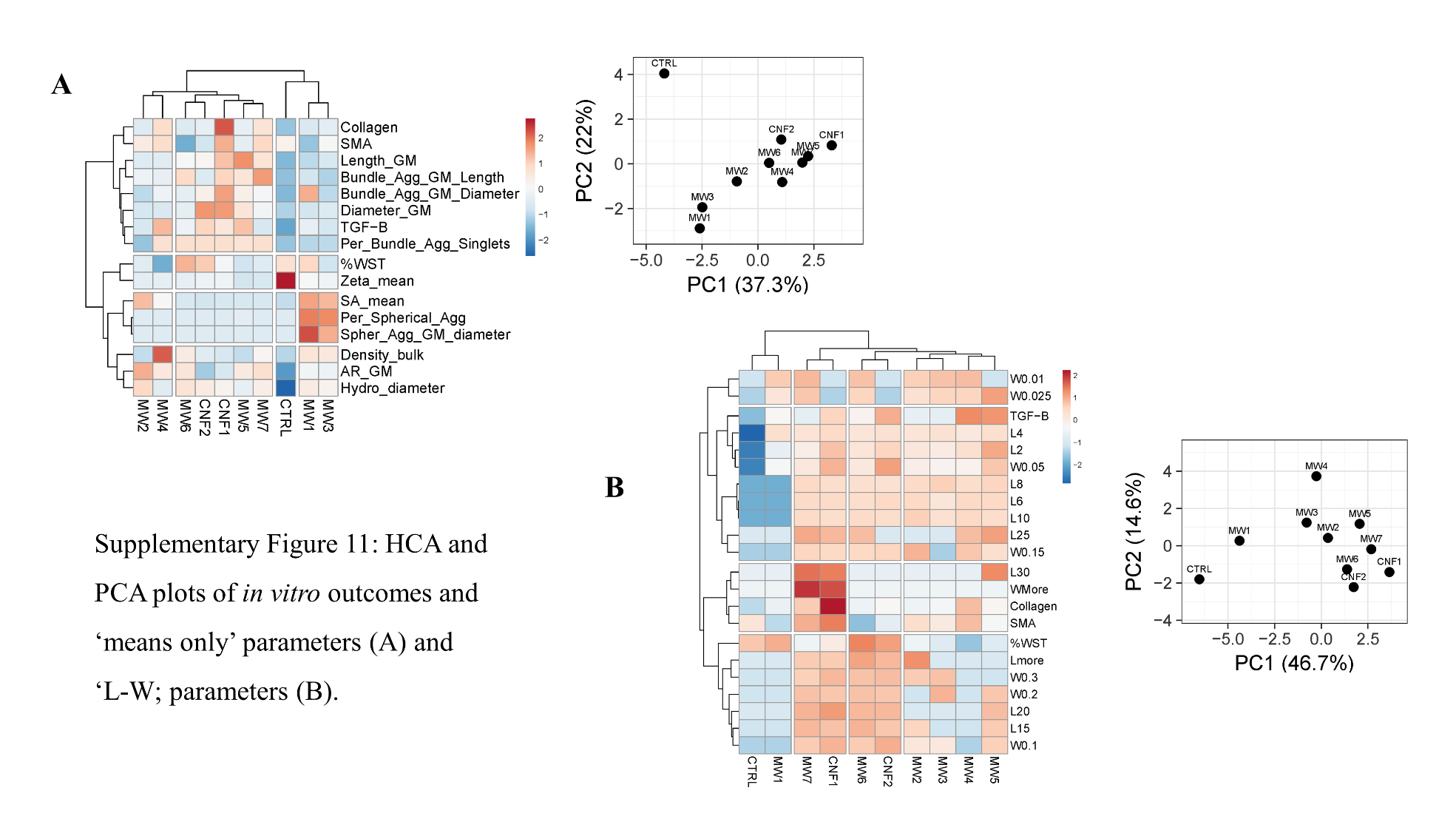

Supplement: Supplementary file 1 — Additional file 1. Supplementary tables and figures. [file 12989_2021_440_MOESM1_ESM.docx]
